# Differential roles of GDF15 and FGF21 in systemic metabolic adaptation to the mitochondrial integrated stress response

**DOI:** 10.1016/j.isci.2021.102181

**Published:** 2021-02-12

**Authors:** Seul Gi Kang, Min Jeong Choi, Saet-Byel Jung, Hyo Kyun Chung, Joon Young Chang, Jung Tae Kim, Yea Eun Kang, Ju Hee Lee, Hyun Jung Hong, Sang Mi Jun, Hyun-Joo Ro, Jae Myoung Suh, Hail Kim, Johan Auwerx, Hyon-Seung Yi, Minho Shong

**Affiliations:** 1 Research Center for Endocrine and Metabolic Diseases, Chungnam National University School of Medicine, 282 Munhwaro, Daejeon 35015, Republic of Korea; 2 Department of Medical Science, Chungnam National University School of Medicine, 266 Munhwaro, Daejeon 35015, Republic of Korea; 3 Center for Research Equipment, Korea Basic Science Institute, Cheongju 28119, Republic of Korea; 4 Convergent Research Center for Emerging Virus Infection, Korea Research Institute of Chemical Technology, Daejeon 34114, Republic of Korea; 5 Graduate School of Medical Science and Engineering, Korea Advanced Institute of Science and Technology, Daejeon 34141, Republic of Korea; 6 Laboratory for Integrative Systems Physiology, Institute of Bioengineering, Ecole Polytechnique Federale de Lausanne, Lausanne 1015, Switzerland; 7 Department of Internal Medicine, Chungnam National University Hospital, Daejeon 35015, Republic of Korea

**Keywords:** Physiology, Cell Biology, Systems Biology

## Abstract

Perturbation of mitochondrial proteostasis provokes cell autonomous and cell non-autonomous responses that contribute to homeostatic adaptation. Here, we demonstrate distinct metabolic effects of hepatic metabokines as cell non-autonomous factors in mice with mitochondrial OxPhos dysfunction. Liver-specific mitochondrial stress induced by a loss-of-function mutation in *Crif1* (LKO) leads to aberrant oxidative phosphorylation and promotes the mitochondrial unfolded protein response. LKO mice are highly insulin sensitive and resistant to diet-induced obesity. The hepatocytes of LKO mice secrete large quantities of metabokines, including GDF15 and FGF21, which confer metabolic benefits. We evaluated the metabolic phenotypes of LKO mice with global deficiency of GDF15 or FGF21 and show that GDF15 regulates body and fat mass and prevents diet-induced hepatic steatosis, whereas FGF21 upregulates insulin sensitivity, energy expenditure, and thermogenesis in white adipose tissue. This study reveals that the mitochondrial integrated stress response (ISR^mt^) in liver mediates metabolic adaptation through hepatic metabokines.

## Highlights

• Hepatic mitoribosomal defect in LKO mice leads to the ISR^mt^ and metabolic reprogramming
• LKO mice have increased insulin sensitivity and are resistant to diet-induced obesity
• GDF15 regulates body and fat mass and prevents hepatic steatosis in LKO mice
• FGF21 improves glucose clearance, energy expenditure, and thermogenesis in LKO mice

## Graphical abstract

**Figure:**
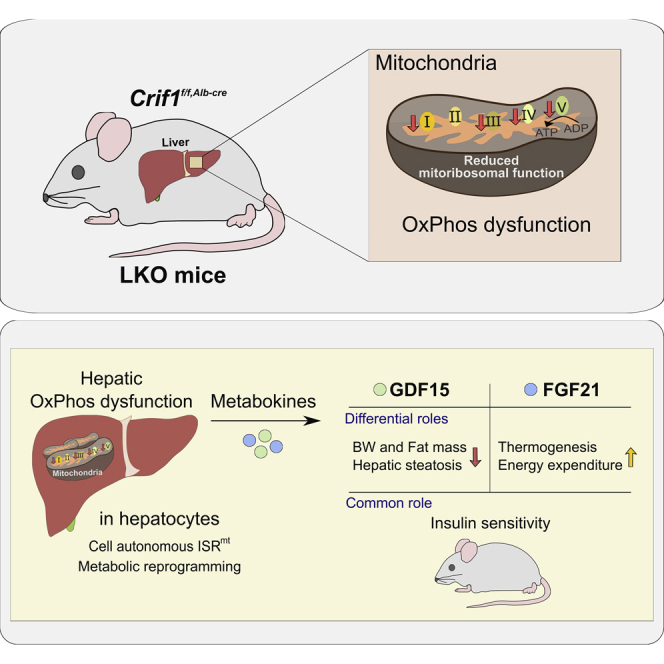

## Introduction

Mitochondria are frequently exposed to conditions of stress, which induce mitochondrial quality control mechanisms that are essential to maintain mitochondrial function (Sorrentino et al., 2018). Cell-autonomous communication from the mitochondria to the nucleus in response to mitochondrial stress has been well-documented in multiple species (Cagin and Enriquez, 2015; Quiros et al., 2017). Concurrently, cell non-autonomous factors are induced by mitochondrial stress, resulting in the communication between tissues that regulate selective gene expression, metabolic reprogramming. and organismal longevity (Durieux et al., 2011; Forsstrom et al., 2019; Kim et al., 2013; Quiros et al., 2016). These elaborated processes have been characterized as a part of the mitochondrial integrated stress response (ISR^mt^), which results from primary defects in mitochondrial DNA replication, the electron transport chain, and mitoribosomal translation (Choi et al., 2020; Chung et al., 2017b; Durieux et al., 2011; Forsstrom et al., 2019; Khan et al., 2017; Martinus et al., 1996; Michel et al., 2015; Quiros et al., 2017).

The liver coordinates systemic metabolism by controlling hepatic metabolic function and regulating metabolism in peripheral tissues. Hepatocyte mitochondria are essential for the maintenance of metabolic plasticity and flexibility (Koliaki and Roden, 2016), and consistent with this, recent studies have shown that mouse models of hepatic mitochondrial dysfunction have alterations in their systemic metabolism, including changes in insulin sensitivity, energy expenditure (EE), and their response to diet-induced obesity (Cho et al., 2017; Kulkarni et al., 2016; Lee et al., 2017; Pospisilik et al., 2007). These findings suggest that the hepatic adaptation to mitochondrial stress may affect systemic energy metabolism through alterations to substrate utilization in the liver and the induction of metabokines that are secreted and can modulate energy metabolism. However, the significance of these hepatic metabokines in the control of systemic metabolism has not been studied extensively.

Growth differentiation factor 15 (GDF15) and fibroblast growth factor 21 (FGF21) are representative metabokines that are responsive to mitochondrial diseases (Kalko et al., 2014; Lehtonen et al., 2016; Lovadi et al., 2017; Montero et al., 2016; Morovat et al., 2017; Yatsuga et al., 2015), and their expression is stimulated through ATF4, CHOP, and XBP1, key components of the integrated stress response (Chung et al., 2017b; Forsstrom et al., 2019; Jiang et al., 2014; Khan et al., 2017; Kim et al., 2013; Patel et al., 2019; Zhang et al., 2018).

These two metabokines have been well-documented as modulators to improve numerous metabolic processes or diseases. One of the representative roles of GDF15 is its anorexigenic effect causing a reduction of body weight, which was first described in mice overexpressing GDF15 (Johnen et al., 2007). A subsequent study showed that mice administered human GDF15 led to increased c-Fos activation in the area postrema and the nucleus of the solitary tract (Tsai et al., 2014). Recently, the GDNF family receptor α-like (GFRAL) has been discovered as a receptor for GDF15 mediating the anorexigenic action, but is localized only in the hindbrain (Emmerson et al., 2017; Hsu et al., 2017; Mullican et al., 2017; Yang et al., 2017). Despite the restriction of GFRAL expression in the hindbrain region of mice, there are numerous studies that cannot simply be explained by the anorexigenic action via GFRAL, including insulin sensitivity, lipolysis in adipose tissue, anti-inflammatory action, alleviation of hepatic steatosis, and muscle atrophy (Chrysovergis et al., 2014; Chung et al., 2017a, 2017b; Garfield et al., 2019; Jung et al., 2018; Zhang et al., 2018).

The physiological role of FGF21 also has an overlapping functional spectrum with GDF15, especially in terms of energy metabolism. FGF21 was described as a novel metabolic regulator enhancing glucose uptake in primary human adipocytes and reducing blood glucose in genetically obese mice administered FGF21 (Kharitonenkov et al., 2005). During fasting or a ketogenic diet, the expression of FGF21 is dependent on the peroxisome proliferator-activated receptor α (PPAR α), which mediates hepatic lipid oxidation, ketogenesis, and gluconeogenesis (Badman et al., 2007, 2009; Inagaki et al., 2007; Potthoff et al., 2009). Moreover, the cross talk between the liver and brain is mediated by FGF21, which regulates glucose homeostasis during prolonged fasting (Liang et al., 2014) and refeeding (Markan et al., 2014), sugar preference in response to carbohydrate intake (von Holstein-Rathlou et al., 2016), and the protein status (Hill et al., 2019). Similar to GDF15, FGF21 reduces the body weight through a non-adipose tissue effect; increases insulin sensitivity, adaptive thermogenesis, and uncoupling protein 1 (UCP1)-independent EE; and reverses hepatic steatosis (BonDurant et al., 2017; Chen et al., 2017; Desai et al., 2017; Hill et al., 2019; Ost et al., 2016; Pereira et al., 2017; Veniant et al., 2015; Xu et al., 2009). However, despite similar metabolic roles and induction mechanism, few studies distinguished between the differing roles of GDF15 and FGF21 in the context of metabolism.

Genetic or pharmacological inhibition of mitochondrial oxidative phosphorylation (OxPhos) results in the activation of cell-autonomous mitochondrial stress signaling, which involves the activation of the mitochondrial unfolded protein response (UPR^mt^) and mitophagy, as well as the secretion of cell non-autonomous factors (Forsstrom et al., 2019; Sorrentino et al., 2018). Mice with a tissue-specific deficiency of *Crif1*, which encodes a protein of the large subunit of the mitochondrial ribosome, have markedly impaired mitoribosome-mediated translation and display many molecular features of the ISR^mt^, including both cell-autonomous and non-autonomous responses (Choi et al., 2020; Chung et al., 2017b; Kim et al., 2012). In the present study, we have explored the effects of hepatic mitochondrial OxPhos dysfunction on energy metabolism in the liver and other key metabolic tissues. We have characterized the adaptive response to mitochondrial OxPhos dysfunction in the liver and investigated its effects on insulin sensitivity and EE. Using double knockout mice (LKO/*Gdf15*^*−/−*^, LGKO and LKO/*Fgf21*^*−/−*^, LFKO), we have identified distinct roles of hepatic GDF15 and FGF21 in response to ISR^mt^. GDF15 regulates body and fat mass and protects against hepatic steatosis, whereas FGF21 increases insulin sensitivity and uncoupling protein (UCP)1-mediated thermogenesis in inguinal adipose tissue (iWAT) in the context of hepatic mitochondrial dysfunction.

## Results

### Aberrant OxPhos by *Crif1* deletion is associated with ISR^mt^ and impairments in insulin signaling and insulin-stimulated glucose uptake in hepatocytes

**Figure 1:**
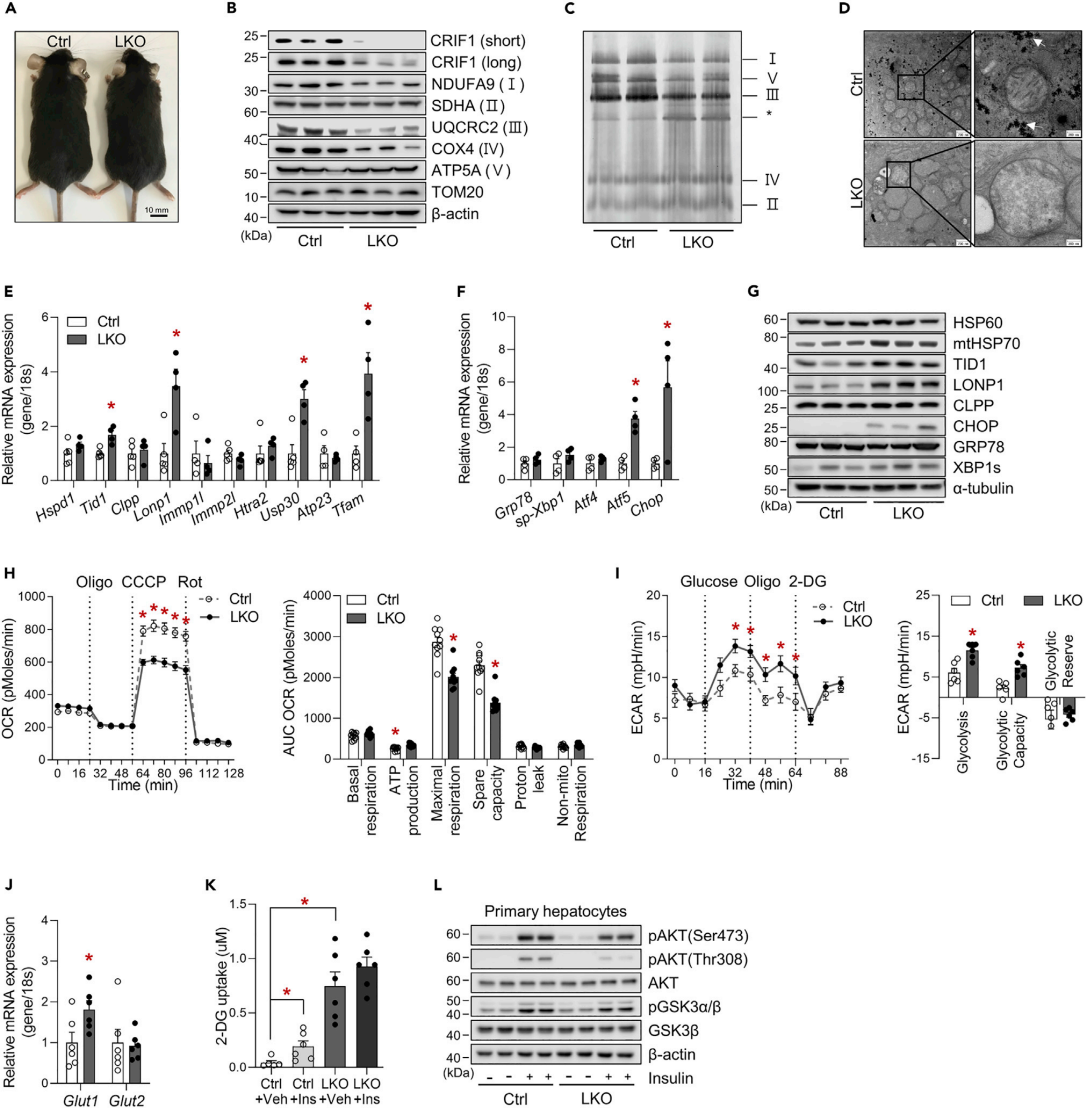
The livers of liver-specific *Crif1*-deficient mice exhibit altered glucose metabolism and impaired insulin signaling (A) Gross morphology of Ctrl and LKO mice at 8 weeks of age. (B) Western blot analysis showing lower levels of CRIF1 and subunits of the OxPhos complex in the livers of Ctrl and LKO mice. The results of one representative experiment of the two conducted are shown. (C) BN-PAGE analysis of the assembled OxPhos complex in the livers of Ctrl and LKO mice (∗: abnormal sub-complexes). (D) Representative transmission electron microscopic images of livers from Ctrl and LKO mice (n = 4). The white arrows indicate hepatic glycogen granules. (E and F) Quantitative PCR analysis of UPR^mt^ mediators (E) and UPR^er^ and transcription factors involved in the mitochondrial stress response (F) in the livers of Ctrl and LKO mice (n = 4–5 biological replicates from three independent experiments). (G) Western blot analysis of UPR^mt^ and UPR^er^ mediators in the livers of Ctrl and LKO mice. The results of one representative experiment out of the three conducted are shown. Data are mean ± SEM and were analyzed using Student's t test (∗p < 0.05 versus Ctrl). (H) OCR (left panel) and individual parameters (right panel) in primary hepatocytes isolated from Ctrl and LKO mice (n = 10 biological replicates from two independent experiments) treated with oligomycin (2 μg/mL), CCCP (10 μM), or rotenone (1 μM). Basal respiration, ATP production, and proton leakage were calculated after oligomycin treatment, and the maximal and non-mitochondrial respiration were calculated after CCCP and rotenone treatment, respectively. (I) Glycolysis assay (left panel) and glycolytic parameters (right panel) in primary hepatocytes isolated from the livers of Ctrl and LKO mice (n = 6 biological replicates from two independent experiments). Non-glycolytic acidification was calculated after the addition of 2-DG (50 mM). Glycolysis, glycolytic capacity, and glycolytic reserve were calculated after the addition of glucose (10 mM) and oligomycin (1 μM), respectively. (J) Quantitative PCR analysis of *Glut1* and *Glut2* mRNA expression in livers from 8-week-old Ctrl and LKO mice (n = 6 biological replicates from three independent experiments). (K) Glucose uptake by primary hepatocytes from Ctrl and LKO mice (n = 6 biological replicates from two independent experiments). Insulin (1 μM) was added 20 min before the measurements. (L) Western blot analysis of insulin signaling after the addition of insulin (200 nM) for 15 min to primary hepatocytes isolated from Ctrl and LKO mice. The results of one representative experiment of the two conducted are shown. Data are mean ± SEM and were analyzed using two-way ANOVA followed by Scheff's post-hoc test in (K) and Student's t test in (E and F) and (H–J) (∗p < 0.05 versus Ctrl or Ctrl-Vehicle). See also Figure S1.

To determine the impact of hepatic mitochondrial OxPhos dysfunction on systemic energy metabolism, we generated liver-specific *Crif1* knockout (LKO) mice through selective disruption of *Crif1* in hepatocytes using the Cre-loxP system (Figures S1A and S1B). The generated mice did not show any obvious abnormalities (Figure 1A). Deficiency of *Crif1* was only present in the liver and resulted in lower expression of OxPhos complex subunits, including NADH:ubiquinone oxidoreductase subunit A9 (NDUFA9), ubiquinol-cytochrome c reductase core protein 2 (UQCRC2), and cytochrome oxidase subunit 4 (COX4) (Figure 1B). Moreover, blue native PAGE (BN-PAGE) showed a reduction in the assembly of OxPhos complexes I, V and III in mitochondria isolated from the livers of LKO mice (Figure 1C). Mitochondria of LKO liver in electron microscopy showed swelling, decreased cristae number, and decreased electron density in the matrix. Hepatocytes from LKO mice contained a small number of glycogen granules (Figure 1D). Histological analysis of the LKO livers revealed enlarged nuclei and abundant cytoplasm, which are histological features of proliferative cells, and an infiltration of mononuclear cells in the centrilobular areas (Figure S1C). Although the serum activity of alanine aminotransferase (ALT) was high in LKO mice (Figure S1D), fluorescence-activated cell sorting analysis suggested that the liver of LKO mice had about a 3-fold increase of infiltrated F4/80^low^CD11b^high^ monocytes and a statistically significant decrease of F4/80^high^CD11b^low^-resident hepatic Kupffer cells. Infiltrated inflammatory cells, CD11b^high^/Ly6G^high^ (neutrophil) and CD11b^high^/LyC^high^ (monocyte), and immunosuppressive cells, CD4^high^CD44^high^CD62L^high^ (Treg), were coincidentally increased in liver of LKO mice (Figure S1E). The hepatic protein levels of apoptosis markers, including cleaved caspase 3 and PARP1, were similar to those of controls (Figure S1F).

*Crif1* deletion induces the accumulation of untranslated OxPhos polypeptides in the mitochondrial matrix due to the abnormal translation of OxPhos subunits (Kim et al., 2012), which initiates the mitochondrial unfolded protein response (UPR^mt^), and consistent with this, the transcripts of the co-chaperone *Tid1*, the intrinsic protease *Lonp1,* and the mitochondrial deubiquitinase *Usp30* were upregulated in the livers of LKO mice (Figure 1E). The expression of C/EBP homologous protein (CHOP) and ATF5, which mediate the UPR^mt^ in a mammalian system (Fiorese et al., 2016; Zhao et al., 2002), were also high, but that of the UPR^er^ regulators *Atf4* and *Grp78* were normal in the livers of LKO mice (Figure 1F). We also found an increase in the hepatic protein levels of mtHSP70, TID1, LONP1, and CHOP in the livers of LKO mice (Figure 1G).

Next, we conducted 2-deoxy-D-glucose (2DG) uptake analysis to determine whether the higher rate of glycolysis is accompanied by higher glucose uptake into hepatocytes (Figures 1H–IJ ). The basal glucose uptake by LKO hepatocytes was much higher than that by control cells, but the effect of insulin on glucose uptake was attenuated in LKO hepatocytes (Figure 1K). Subsequently, we measured insulin-stimulated AKT phosphorylation in cultured hepatocytes and found significantly lower insulin-induced phosphorylation of AKT at Ser473 and Thr308 in primary LKO hepatocytes than in control hepatocytes (Figure 1L). Taken together, these findings imply that hepatic OxPhos dysfunction in LKO mice is associated with activation of ISR^mt^ and lower insulin-stimulated AKT activation, but greater glycolysis and glucose uptake.

### Defective hepatic OxPhos is associated with higher systemic insulin sensitivity and energy expenditure

**Figure 2:**
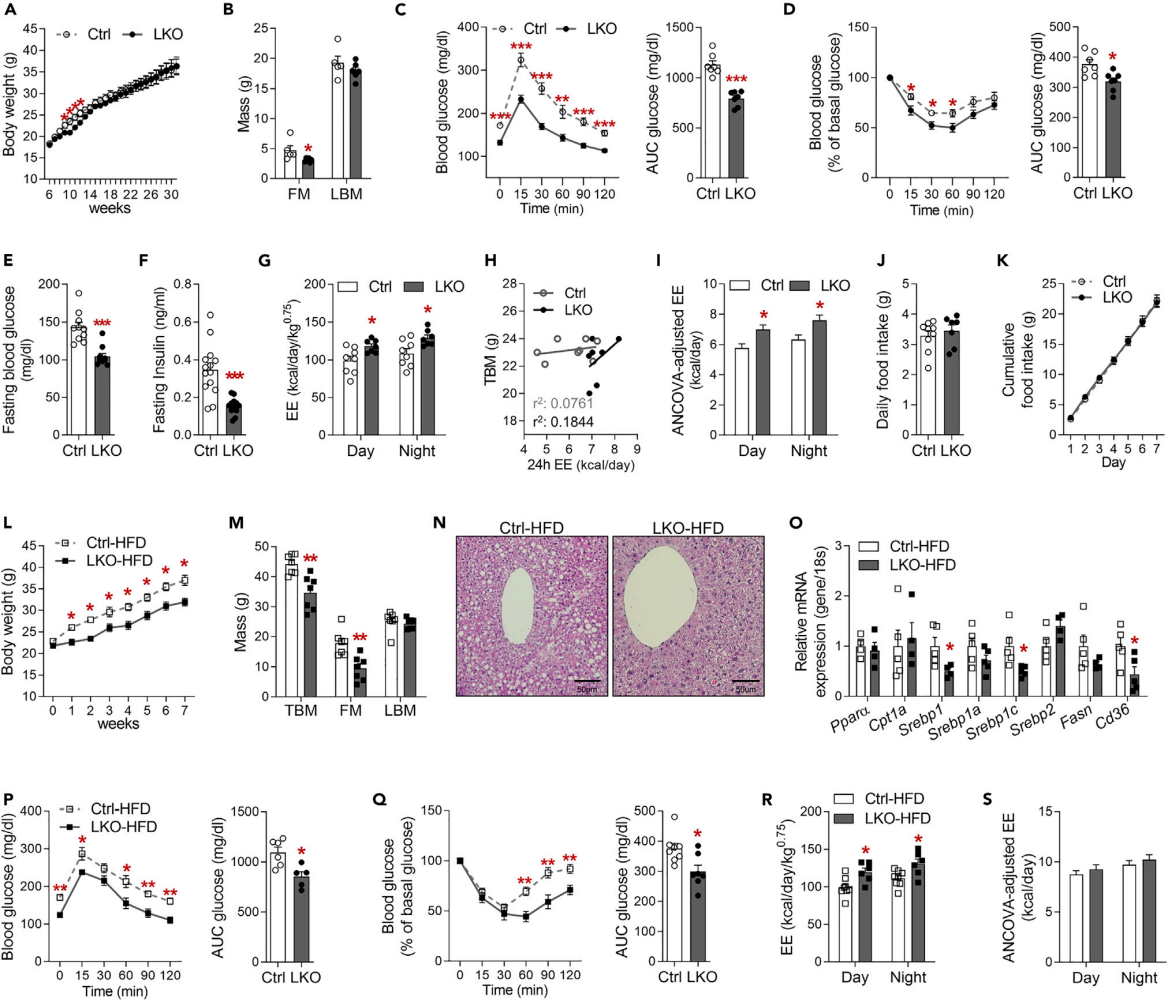
Liver-specific *Crif1*-deficient mice exhibit superior energy metabolism and are protected against diet-induced obesity Mice fed a chow diet were used in (A–K) and mice fed an HFD for 8 weeks were used in (L–S). (A) Body masses of Ctrl and LKO mice from 6 to 31 weeks of age (n = 10 biological replicates from two independent experiments). (B) Body composition of Ctrl and LKO mice (n = 5–6 biological replicates from two independent experiments). (C) Glucose tolerance test (GTT) (left panel) and glucose area under the curve (AUC) (right panel) for Ctrl and LKO mice (n = 7 biological replicates from three independent experiments). (D) Insulin tolerance test (ITT) (left panel) and glucose area under the curve (AUC) (right panel) for Ctrl and LKO mice (n = 7 biological replicates from three independent experiments). (E) Blood glucose concentrations of Ctrl and LKO mice fasted for 6 h (n = 10 biological replicates from five independent experiments). (F) Serum insulin concentrations in Ctrl and LKO mice fasted for 6 h (n = 10–12 biological replicates from three independent experiments). (G) Measurement of energy expenditure (EE) per metabolic body mass of Ctrl and LKO mice (n = 7–8 biological replicates). (H and I) Correlation analysis between EE and total body mass (TBM) (H) and ANCOVA-adjusted EE (I) in Ctrl and LKO mice. (J and K) Daily food intake (J) and cumulative food intake over 7 days (K) in mice (n = 7–9 biological replicates from five experiments). (L) Body mass (n = 5 biological replicates from three independent experiments). (M) Body composition in Ctrl and LKO mice (n = 7 biological replicates from two independent experiments). (N) Representative images of H&E-stained liver sections, showing a central vein, from Ctrl and LKO mice (n = 5–6 biological replicates). (O) Quantitative PCR analysis of the expression of genes involved in lipid metabolism in the livers of Ctrl and LKO mice (n = 4–5 biological replicates from two independent experiments). (P) Glucose tolerance test (GTT, left panel) and glucose area under the curve (AUC, right panel) for Ctrl and LKO mice (n = 5 biological replicates from three independent experiments). (Q) Insulin tolerance test (ITT, left panel) and glucose area under the curve (AUC, right panel) for Ctrl and LKO mice (n = 5 biological replicates from three independent experiments). (R and S) EE per metabolic body mass (R) and ANCOVA-adjusted EE (S) in control and LKO mice (n = 7 biological replicates). Data are mean ± SEM. Statistical analyses were performed using Student's t test in (A–G), (J–M), and (O–R) or ANCOVA in (I) and (S) (∗p < 0.05, ∗∗p < 0.01, ∗∗∗p < 0.001 versus Ctrl). See also Figure S2.

Chow-fed LKO mice exhibited similar body masses to control mice at 30 weeks of age (Figure 2A). Although LKO hepatocytes were characterized by large nuclei and cytoplasmic volumes, the liver mass was similar to that of control mice, whereas the fat mass of LKO mice was significantly lower than that of controls (Figures 2B and S2A). To determine whether poor hepatic OxPhos function affects systemic insulin sensitivity, we conducted glucose or insulin tolerance testing in mice fed a chow diet at 8 weeks of age. Interestingly, the glucose and insulin tolerance of LKO mice was superior to those of control mice (Figures 2C and 2D), which translated into lower blood glucose and serum insulin concentrations (Figures 2E and 2F). Alongside the lower basal blood glucose levels in LKO mice, the fasting-induced expression of glucose 6-phosphatase (*G6pase*) and phosphoenolpyruvate carboxykinase (*Pepck*), which are the rate-limiting enzymes in gluconeogenesis, was also lower in LKO mice than in controls (Figure S2B). Furthermore, the homeostatic model assessment for insulin resistance (HOMA-IR) suggested that LKO mice had significantly higher systemic insulin sensitivity than controls (Figure S2C), despite the disruption to hepatic insulin signaling. The serum triglyceride concentration of LKO mice was similar to that of controls, but the total cholesterol concentration was lower (Figures S2D and S2E).

To explore the effects of hepatic OxPhos dysfunction on systemic EE, we measured O_2_ consumption and CO_2_ production by indirect calorimetry. LKO mice had a higher EE/metabolic body mass (body mass^0.75^) ratio than control mice (Figure 2G). Plots of individual data and the ANCOVA-adjusted EE with body mass as a covariate revealed that LKO mice had significantly higher EE than controls, even after the effects of mass were eliminated (Figures 2H and 2I). The factors that influence total EE in the mice were then assessed by analyzing locomotor activity (Figure S2F), body temperature (Figure S2G), and food intake (Figures 2J and 2K), which were all similar in LKO and control mice. LKO mice had a tendency toward a lower respiratory quotient (RQ) than control mice (Figure S2H), but there was no difference in the serum concentration of the ketone body β-hydroxybutyrate (BHB) (Figure S2I). Taken together, these data suggest that hepatic OxPhos dysfunction is associated with higher systemic insulin sensitivity and EE, but not differences in body temperature or food intake, even though insulin signaling and gluconeogenesis are impaired in the livers of LKO mice.

### LKO mice are protected against diet-induced obesity and insulin resistance

To determine whether hepatic OxPhos dysfunction alleviates metabolic stress, LKO mice and controls were fed a high-fat diet (HFD) for 8 weeks from 6 weeks of age. LKO mice were already gaining significantly less body mass than controls after 1 week of HFD feeding (Figure 2L). After 8 weeks, the masses of iWAT, epididymal white adipose tissue (eWAT), and brown adipose tissue (BAT) in LKO mice were lower than those of controls (Figure S2J). Thus, the lower body mass of LKO mice fed an HFD was likely the result of resistance to fat accumulation (Figure 2M). LKO mice also showed lower hepatic lipid accumulation (Figure 2N), which was associated with lower hepatic expression of *Srebp1, Srebp1c,* and *Cd36* (Figure 2O). The glucose and insulin tolerance of LKO mice fed an HFD were higher than those of control mice on the same diet (Figures 2P and 2Q), which was consistent with the lower blood glucose, insulin, and HOMA-IR in the fasted LKO mice (Figures S2K, S2L, and S2M). The serum concentrations of triglyceride were similar in LKO mice and controls (Figure S2N), but the total cholesterol concentration was lower in HFD-fed LKO mice (Figure S2O).

Next, we assessed the EE of the mice by indirect calorimetry. When consuming an HFD, the EE adjusted for metabolic body mass was significantly higher in LKO than in control mice during both the night and daytime (Figure 2R), but the ANCOVA-adjusted EE was similar in the two genotypes (Figure 2S). RQ tended to be lower in LKO mice (Figure S2P), but the serum BHB concentrations were similar in the two groups (Figure S2Q). Taken together, these results demonstrate that LKO mice are resistant to diet-induced obesity and protected against hepatic steatosis and insulin resistance when fed an HFD.

### The adipose tissue of LKO mice exhibits enhanced insulin signaling and fatty acid oxidation

**Figure 3:**
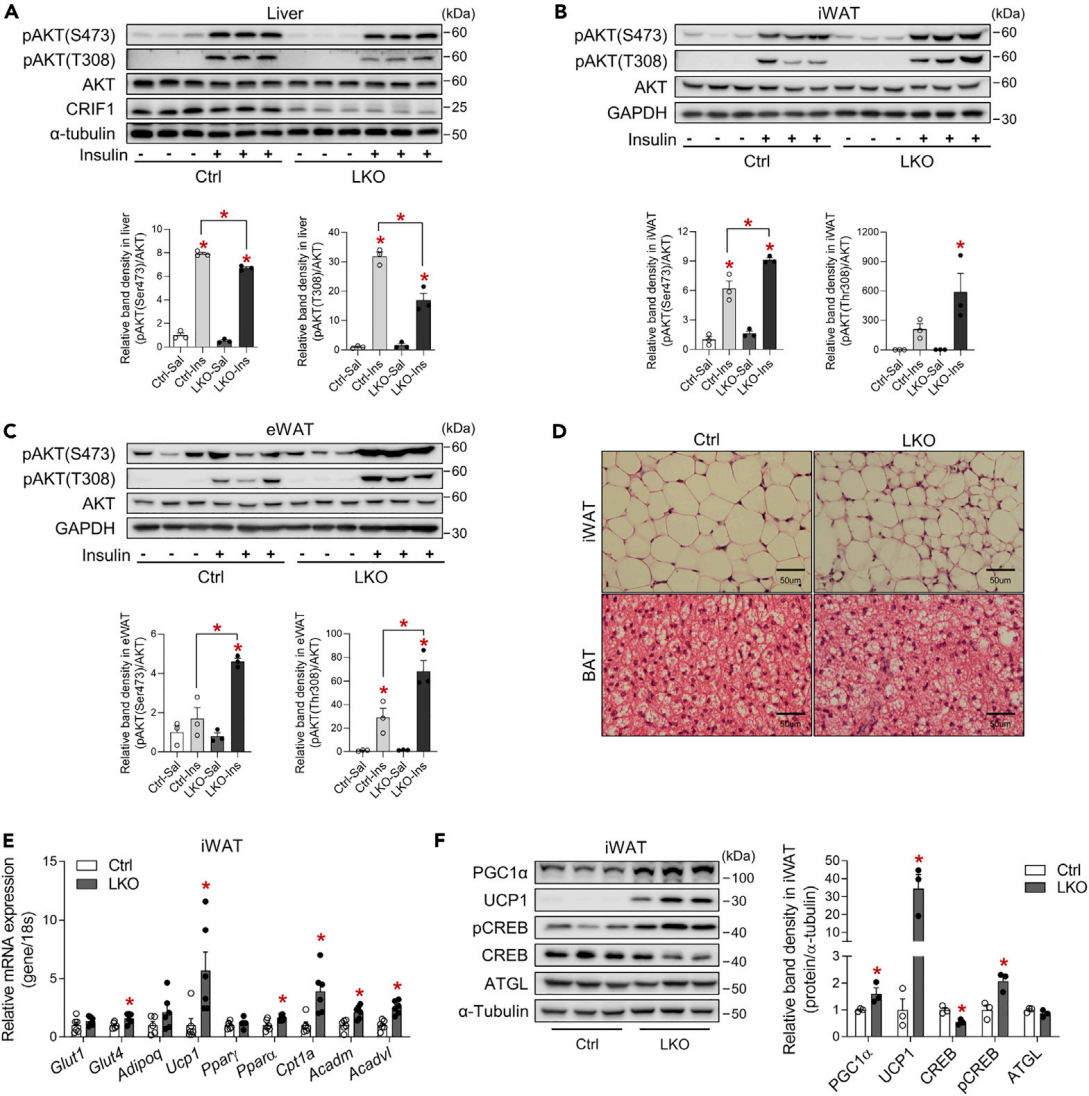
The white adipose tissue of liver-specific *Crif1*-deficient mice exhibits greater insulin signaling and fatty acid metabolism (A–C) Western blot analysis of AKT phosphorylation in the liver (A), iWAT (B), and eWAT (C) of Ctrl and LKO mice (n = 3 biological replicates per group). Mice were fasted for 6 h and then administered intraperitoneally with insulin (4 U/kg). One representative experiment of the two conducted is shown. (D) Representative images of hematoxylin and eosin-stained iWAT and BAT sections from Ctrl and LKO mice (n = 4 biological replicates). (E) Quantitative PCR analysis of the expression of genes involved in glucose uptake and fatty acid metabolism in the iWAT of Ctrl and LKO mice (n = 6 biological replicates from two independent experiments). (F) Western blot analysis of signaling upstream of UCP1 in the iWAT of Ctrl and LKO mice (n = 3 biological replicates). The results of one representative experiment of the three conducted are shown. Mice were fed a chow diet. Data are mean ± SEM and were analyzed using two-way ANOVA followed by Scheff's post-hoc test in (A–C) and Student's t test in (E) and (F) (∗p < 0.05 versus Ctrl or Ctrl-Saline). See also Figure S3.

To assess the insulin sensitivity of key metabolic tissues in LKO mice, we evaluated insulin signaling and action in the liver, iWAT, eWAT, BAT, and gastrocnemius muscle (GM) after the intraperitoneal injection of insulin (4 U/kg). AKT phosphorylation at Ser473 and Thr308 was significantly lower in the livers of LKO mice than controls (Figure 3A), but insulin-stimulated AKT phosphorylation at Ser473 and Thr308 was much higher in both the iWAT and eWAT of LKO mice (Figures 3B and 3C). However, the AKT phosphorylation in GM was similar in LKO and control mice, but the AKT phosphorylation at Ser 473 in BAT was decreased in LKO mice (Figures S3A and S3B).

LKO mice exhibited cellular heterogeneity in their iWAT, but not BAT, upon histological analysis (Figure 3D). Moreover, the expression of *Glut4*, *Pparα*, *Cpt1a*, and acyl-CoA dehydrogenase medium chain (*Acadm*) and very long chain (*Acadvl*) were significantly increased in the iWAT (Figure 3E), but not in the eWAT, BAT, or GM of LKO mice (Figures S3C and S3D). Interestingly, UCP1 expression was elevated in the iWAT and eWAT of LKO mice. Furthermore, the phosphorylation of CREB at Ser133 was significantly increased in the iWAT of LKO mice (Figure 3F). This is consistent with the induction of adipose UCP1 expression in LKO mice. Taken together, these data suggest that hepatic OxPhos dysfunction is associated with enhanced insulin signaling and fatty acid oxidation in adipose tissue depots.

### Transcriptome analysis identifies altered biological pathways and transcripts associated with adaptive mitochondrial stress in LKO mice

**Figure 4:**
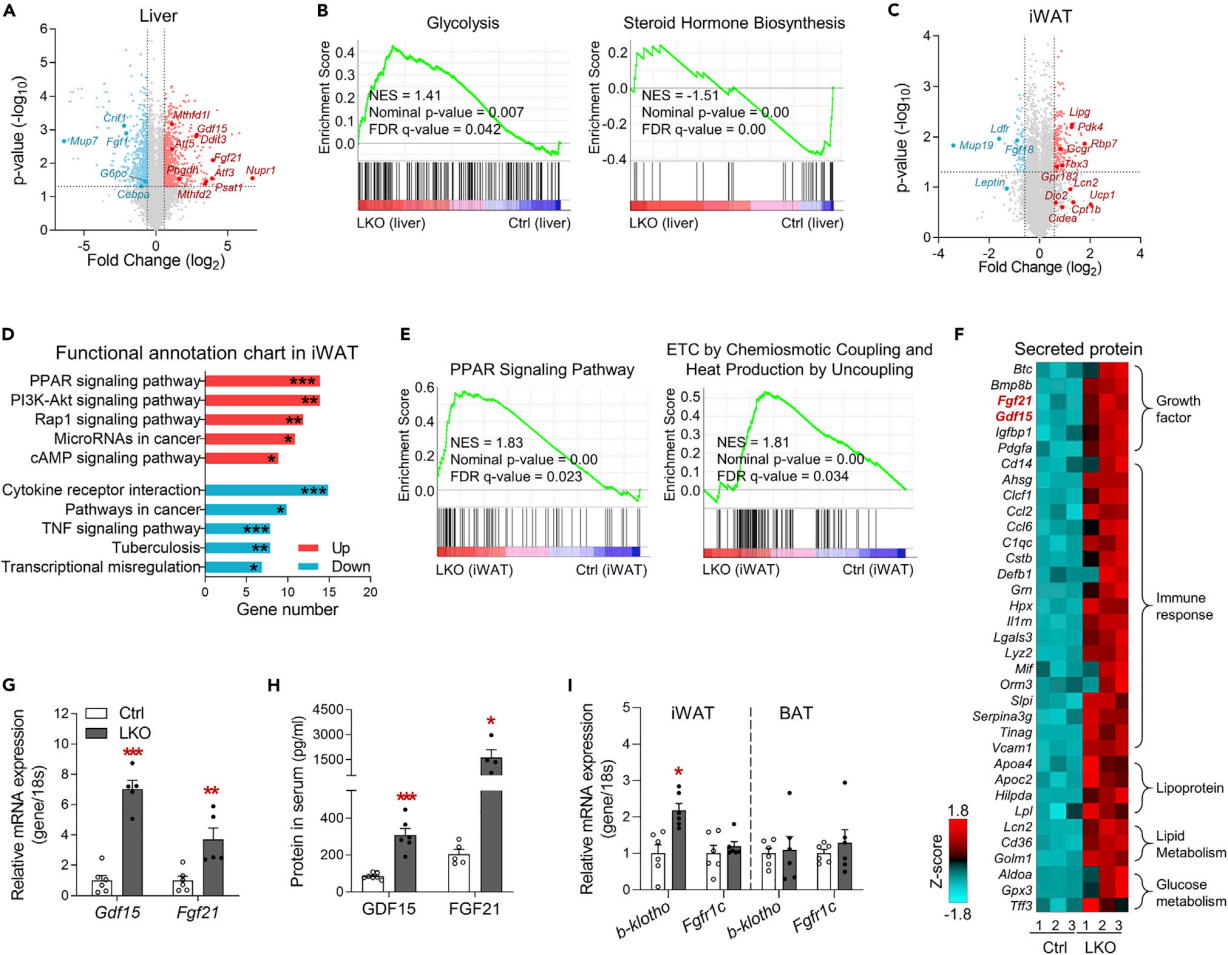
Liver-specific *Crif1* deficiency increases the production of metabokines in liver (A) Volcano plot showing the DEGs in the liver of LKO mice. The colored dots indicate the DEGs with ≥ ±1.5-fold difference from Ctrl mice. The red and blue dots indicate the upregulated and downregulated transcripts, respectively. (B) Gene set enrichment analysis (GSEA) using DEGs in the liver of Ctrl and LKO mice. (C and D) Volcano plot showing the DEGs (C) and top-ranked functional annotation chart (D) in the iWAT of LKO mice. Functional annotation was categorized using the KEGG pathway in DAVID (ver.6.8), and the results are ordered according to gene number. (E) GSEA showing the upregulated gene set in the iWAT of LKO mice. (F) Heatmap of the DEGs classified as “Secreted proteins” in the cellular compartment category (≥2-fold difference, p < 0.05 versus Ctrl mice). The genes in red were the two top-ranked transcripts, *Fgf21* and *Gdf15* in the liver of LKO mice. (G) Quantitative PCR analysis of *Gdf15* and *Fgf21* mRNA expression in Ctrl and LKO livers (n = 5–6 biological replicates from four independent experiments). (H) Serum GDF15 (n = 6–7 biological replicates) and FGF21 (n = 4–5 biological replicates) in Ctrl and LKO mice. Three independent experiments were performed. (I) Quantitative PCR analysis of *Klb* and *Fgfr1c* mRNA expression in adipose tissue of mice (n = 6 biological replicates). The mice were studied at 8–9 weeks of age and fed a chow diet. Data in (G–I) are mean ± SEM. Statistical analyses were performed using Student's t test in (G–I) (∗p < 0.05, ∗∗p < 0.01, ∗∗∗p < 0.001 versus Ctrl). The data in (D) were analyzed using a modified Fisher's exact p value (∗p < 0.05, ∗∗p < 0.01, ∗∗∗p < 0.001). Un.d, undetectable. See also Figure S4.

To better understand the effects of hepatic OxPhos dysfunction on systemic energy metabolism, multi-tissue transcriptomic analysis was performed on the liver, iWAT, GM, and hypothalamus of the mice. A Venn diagram shows the number of transcripts with a significant > ±1.5-fold difference in the liver (1,325), iWAT (251), GM (36), and hypothalamus (17) of LKO mice (Figures S4A and S4B, and Table S2). Pyruvate dehydrogenase kinase 4 (*Pdk4*), switching the glucose catabolism to fatty acid utilization, and alkaline ceramidase 2 (*Acer2*), responding to DNA damage, were commonly increased in liver, iWAT, and GM of LKO mice. The livers of LKO mice exhibited higher expression of transcripts related to the ISR^mt^, including that of transcripts encoding transcription factors (*Nupr1*, *Atf3,* and *Atf5*), hepatic metabokines (*Gdf15* and *Fgf21*), and proteins involved in single-carbon (1C) metabolism (*Mthfd1l* and *Mthfd2*) (Figure 4A). Gene set enrichment analysis (GSEA) suggested that *Crif1* deficiency activated glycolysis (Figure 4B, left panel). Functional annotation charts based on the KEGG pathway also indicated that gene sets of glutamine, 1C, glycogen, and fatty acid metabolism were increased in the liver of LKO mice (Figures S4C and S4D, and Table S3), whereas steroid hormone biosynthesis and transcription factors involved in lipid metabolism were downregulated in LKO livers (Figures 4B, right panel, and S4E).

The transcripts associated with browning (*Ucp1*, *Dio2*, *Cidea,* and *Cox7a1*) and fatty acid oxidation (*Pparα*, *Cpt1b,* and *Cd36*) were upregulated in the iWAT of LKO mice (Figure 4C). The functional annotation chart and GSEA in iWAT revealed that gene sets related to PPAR, PI3K-AKT, and the cyclic adenosine 3′,5′-monophosphate (cAMP) signaling pathways were significantly enriched in the LKO mice (Figures 4D and 4E, and Table S3), which was confirmed by an increase in insulin signaling in the WAT of LKO mice (Figures 3B and 3F).

Cell non-autonomous factors induced during the ISR^mt^ play a key role in inter-organ communication and the reprogramming of systemic energy metabolism. We therefore analyzed the differentially expressed genes (DEGs) termed as secreted proteins in liver and found that transcripts classified as growth factor, immune response, lipoprotein, and mediators related with lipoid and glucose metabolism were associated with defective OxPhos in liver (Figure 4F). Among the transcripts that have known to improve the insulin sensitivity, *Fgf21* (15.5-fold change) and *Gdf15* (7.2-fold change) were mostly increased in the liver of LKO mice with significance. Mitochondrial OxPhos dysfunction increased not only hepatic *Gdf15* and *Fgf21* mRNA expression but also the serum concentrations of GDF15 and FGF21 in the LKO mice (Figures 4G and 4H). In addition, the expression of β-klotho, a co-receptor for FGF21, was also high in the iWAT of LKO mice, which might be because of greater effects of FGF21 (Figure 4I). Taken together, these data show that mitochondrial OxPhos dysfunction in the liver alters the hepatic transcriptome involved in coordinating the ISR^mt^ (including cell-autonomous and non-cell autonomous factors), and these hepatic changes were associated with greater insulin action in adipose tissues and the regulation of systemic energy homeostasis.

### Distinct roles of the hepatic metabokines GDF15 and FGF21 in systemic energy metabolism

Although the physiological role of GDF15 and FGF21 have been well established through respective studies using pharmacological and genetic approaches, it is still unclear how concurrently induced metabokines differently act and which is more potential to improve the metabolism.

Therefore, to investigate the roles of these major hepatic metabokines in LKO mice, we generated LKO mice with global *Gdf15* deletion (LGKO) or global *Fgf21* deletion (LFKO) on a C57BL/6 background (Figures S5A and S5B). LGKO and LFKO mice exhibited much lower expression of *Gdf15* and *Fgf21*, respectively, than control mice (Figures S5C and S5D). Interestingly, deficiency of one of these metabokines did not affect the expression of the other metabokine in LKO mice (Figures S5E and S5F).

**Figure 5:**
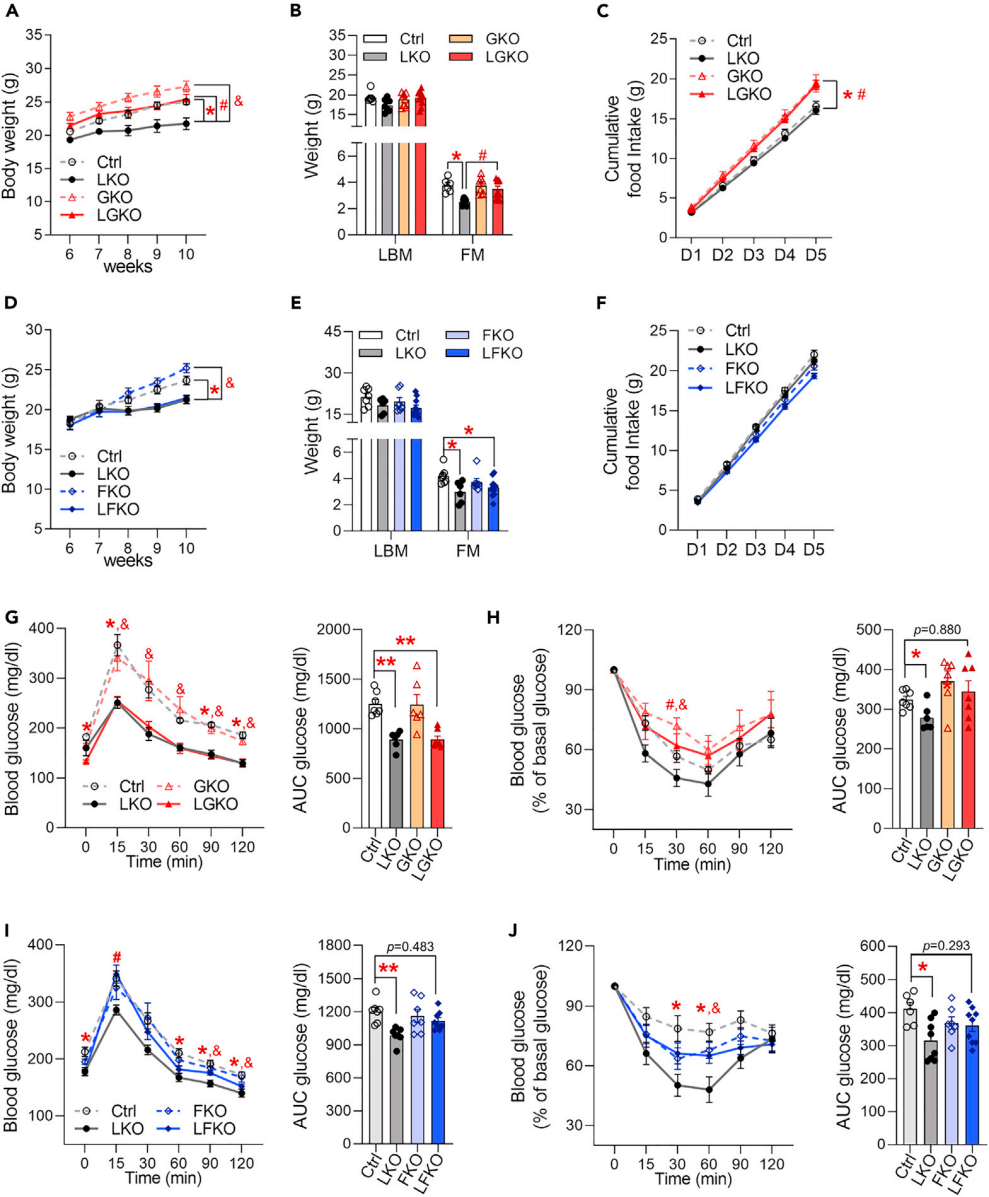
GDF15 regulates body and fat mass, and FGF21 regulates glucose clearance in liver-specific *Crif1*-deficient mice fed a chow diet (A) Body masses of Ctrl, LKO, GKO, and LGKO mice (n = 7 biological replicates from five independent experiments). (B) Body composition, measured by DXA, of Ctrl, LKO, GKO, and LGKO mice (n = 7–11 biological replicates from three independent experiments). (C) Cumulative food intake over 5 days in Ctrl, LKO, GKO, and LGKO mice housed in individual cages (n = 7–11 biological replicates). (D) Body masses of Ctrl, LKO, FKO, and LFKO mice (n = 8–12 biological replicates from two independent experiments). (E) Body composition, measured by DXA, of Ctrl, LKO, FKO, and LFKO mice (n = 7–10 biological replicates from two independent experiments). (F) Cumulative food intake over 5 days in Ctrl, LKO, FKO, and LFKO mice (n = 8–12 biological replicates from two independent experiments). (G) Glucose tolerance test (left panel) and glucose area under the curve (AUC, right panel) after fasting for 6 h in Ctrl, LKO, GKO, and LGKO mice (n = 6–7 biological replicates per group from three independent experiments). (H) Insulin tolerance test (left panel) and glucose area under the curve (AUC, right panel) in Ctrl, LKO, GKO, and LGKO mice fasted for 6 h (n = 6–7 biological replicates per group from three independent experiments). (I) Glucose tolerance test (left panel) and glucose area under the curve (right panel) in Ctrl, LKO, FKO, and LFKO mice fasted for 6 h (n = 7–8 biological replicates from three independent experiments). (J) Insulin tolerance test (left panel) and glucose area under the curve (right panel) in Ctrl, LKO, FKO, and LFKO mice fasted for 6 h (n = 7–8 biological replicates from three independent experiments). The mice were studied at 8–10 weeks of age and fed a chow diet. Data are expressed as the mean ± SEM and were analyzed by ANOVA followed by Scheff's post-hoc test (∗p < 0.05 versus Ctrl, ∗∗p<0.001, #p < 0.05 for LKO versus DKO, &p < 0.05 for LKO versus GKO or FKO). See also Figure S5.

Next, we compared the effects of the two hepatic metabokines on systemic energy homeostasis. LGKO mice had higher body and fat mass than LKO mice (Figures 5A and 5B). Although the ablation of GDF15 increased the food intake of both the GKO and LGKO mice, LKO mice (with endogenous plasma concentrations of GDF15 of 308±88.04 pg/mL) consumed similar amounts of food as controls (Figure 5C). By contrast, LFKO mice exhibited similar body and fat mass gains to LKO mice (Figures 5D and 5E) as well as food intake (Figure 5F). glucose tolerance test (GTT) revealed that glucose clearance was better in LKO and LGKO mice than in controls (Figure 5G), but LGKO mice showed an intermediate glucose disposal rate during insulin tolerance test (ITT) (Figure 5H), which suggests that GDF15 affects insulin-stimulated glucose disposal. By contrast, LFKO mice showed high blood glucose concentrations 15 min after glucose challenge and intermediate insulin tolerance, when compared with LKO mice, which indicates that FGF21 affects acute glucose clearance and disposal (Figures 5I and 5J). Taken together, these data suggest that hepatic GDF15 secretion in the context of the ISR^mt^ regulates body and fat mass, regardless of food intake, whereas hepatic FGF21 has a glucose-lowering effect. Thus, both metabokines influence insulin-stimulated glucose disposal.

### Fgf21 deficiency is associated with less browning and lower systemic energy expenditure in LKO mice

**Figure 6:**
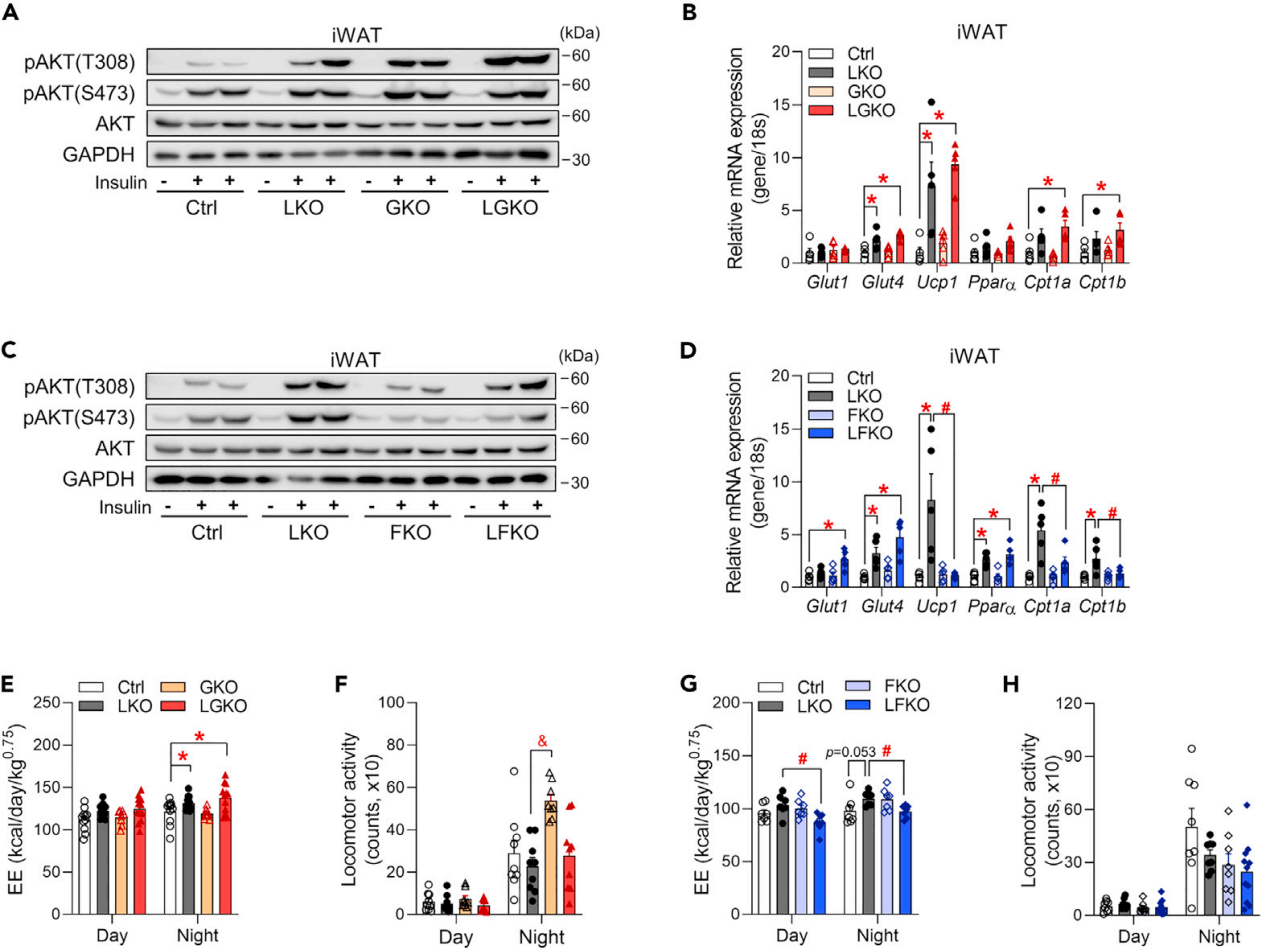
FGF21 increases insulin signaling and the expression of fatty acid metabolic genes in the iWAT of liver-specific *Crif1*-deficient mice (A) Western blot analysis of AKT phosphorylation in the iWAT of Ctrl, LKO, GKO, and LGKO mice. Mice were fasted for 6 h and then injected intraperitoneally with insulin (4 U/kg). The results of one representative experiment of the two conducted are shown. (B) Quantitative PCR analysis of genes involved in glucose transport and fatty acid oxidation in the iWAT of Ctrl, LKO, GKO, and LGKO mice (n = 5 biological replicates from two independent experiments). (C) Western blot analysis of AKT phosphorylation in the iWAT of Ctrl, LKO, FKO, and LFKO mice. Mice were fasted for 6 h and then injected intraperitoneally with insulin (4 U/kg). The results of one representative experiment of the two conducted are shown. (D) Quantitative PCR analysis of genes involved in glucose transport and fatty acid oxidation in the iWAT of Ctrl, LKO, FKO, and LFKO mice (n = 5 biological replicates from two independent experiments). (E) EE per metabolic body mass (BM^0.75^) in Ctrl, LKO, GKO, and LGKO mice (n = 8–12 biological replicates). After 2 days of acclimatization, EE was measured for 2 days. (F) Locomotor activity in Ctrl, LKO, GKO, and LGKO mice housed in individual cages (n = 8–9 biological replicates). Activity was monitored after 1 day of acclimatization. (G) EE per metabolic body mass (BM^0.75^) in Ctrl, LKO, FKO, and LFKO mice (n = 7 biological replicates). (H) Locomotor activity in Ctrl, LKO, FKO, and LFKO mice housed in individual cages (n = 8–11 biological replicates). Data are expressed as the mean ± SEM and were analyzed by ANOVA followed by Scheff's post-hoc test (∗p < 0.05 versus Ctrl, #p < 0.05 for LKO versus DKO, &p < 0.05 for LKO versus GKO or FKO). See also Figure S6.

Next, to dissect the mechanism whereby hepatic metabokines affect insulin signaling in the WAT and the EE of LKO mice, we measured the insulin-induced AKT phosphorylation in the iWAT of double knockout mice. LGKO mice showed consistently an increase in insulin-induced AKT phosphorylation at Thr308 in iWAT (Figures 6A and S6A). Moreover, the high expression of *Glut4*, *Ucp1,* and *Cpt1a/b* that was identified in the iWAT of LKO mice remained in LGKO mice (Figure 6B), whereas LFKO mice showed a decrease in both phosphorylation at Thr308 and Ser473 in iWAT following intraperitoneal insulin injection (Figures 6B, 6C, and S6B). Interestingly, the expression of *Ucp1* and *Cpt1 a/b* was significantly lower in LFKO mice than in LKO mice (Figure 6D). EE was significantly higher in LGKO mice than in control mice during the night (Figure 6E), even though the physical activity of the two groups was similar (Figure 6F). By contrast, the high EE in LKO mice (day, p = 0.1385; night, p = 0.0535) was significantly reduced in LFKO mice, despite similar physical activity (Figures 6G and 6H), which suggests that FGF21 is required for the high EE in LKO mice. Taken together, these data suggest that FGF21 increases not only insulin signaling and EE but also the expression of genes involved in energy metabolism in the iWAT. However, GDF15 is involved the glucose disposal mediated by insulin, not required for the improvements in energy homeostasis in mice that have aberrant hepatic OxPhos.

### GDF15 contributes to the prevention of hepatic steatosis in LKO mice fed a high-fat diet

**Figure 7:**
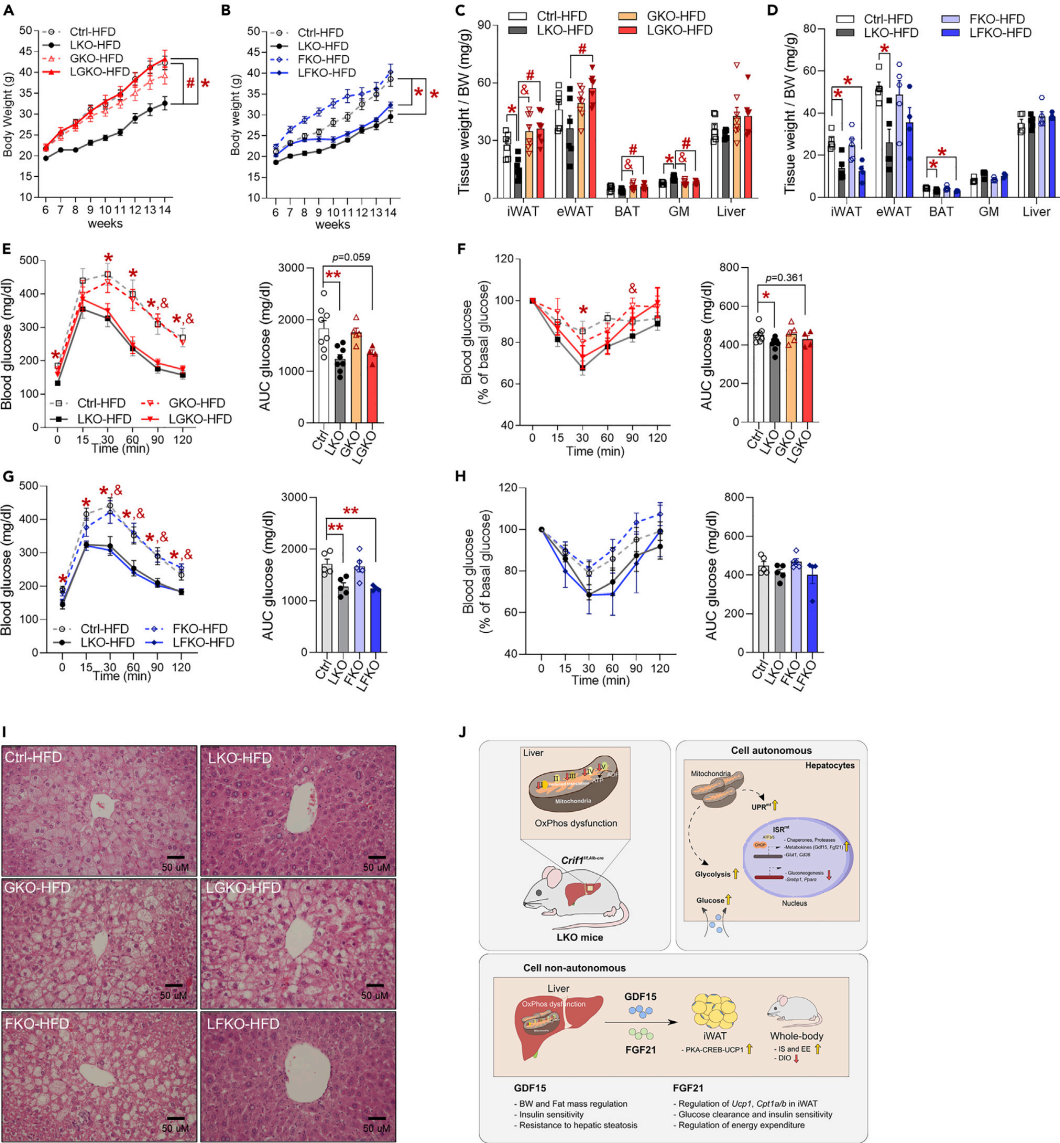
Genetic ablation of *Gdf15* in LKO mice worsens hepatic steatosis, independent of insulin sensitivity (A) Body masses of Ctrl, LKO, GKO, and LGKO mice fed a high-fat diet (60% fat) from 6 to 14 weeks of age (n = 7 biological replicates from two independent experiments). (B) Body masses of Ctrl, LKO, FKO, and LFKO mice fed a high-fat diet from 6 to 14 weeks of age (n = 6–8 biological replicates from two independent experiments). (C) Tissue masses per unit body mass of Ctrl, LKO, GKO, and LGKO mice fed a high-fat diet (n = 7–8 biological replicates from two independent experiments). (D) Tissue masses per unit body mass of Ctrl, LKO, FKO, and LFKO mice fed a high-fat diet (n = 5 biological replicates from two independent experiments). (E and F) Glucose tolerance (E) and insulin tolerance test (F) data for Ctrl, LKO, GKO, and LGKO mice fed a high-fat diet (n = 5–8 biological replicates from two independent experiments). (G and H) Glucose tolerance (G) and insulin tolerance test (H) data for Ctrl, LKO, FKO, and LFKO mice fed a high-fat diet (n = 5 biological replicates from two independent experiments). (I) Representative hematoxylin and eosin-stained liver sections from Ctrl, LKO, GKO, LGKO, FKO, and LFKO mice fed a high-fat diet for 8 weeks (n = 4–5 biological replicates). (J) A schematic model showing consequence upon hepatic ISR^mt^ and action of GDF15 and FGF21 in those mice. All data are expressed as the mean ± SEM and were analyzed using ANOVA followed by Scheff's post-hoc test (∗p < 0.05 versus Ctrl, #p < 0.05 for LKO versus DKO, &p < 0.05 for LKO versus GKO or FKO).

To verify whether hepatic metabokines confer resistance to diet-induced obesity, all the groups of mice were fed an HFD for 8 weeks. LGKO mice showed greater body mass gain than LKO mice, but that of the LFKO mice was similar to that of the LKO mice when consuming an HFD (Figures 7A and 7B). The masses of the iWAT, eWAT, and BAT depots were much higher in LGKO mice than in LKO mice, but *Fgf21* deficiency did not alter the masses of adipose tissue depots in LKO mice (Figures 7C and 7D), which suggests that GDF15 influences the body and fat mass of LKO mice fed an HFD.

The glucose clearance during GTT by HFD-fed LGKO and LFKO mice was similar to that of LKO mice (Figures 7E and 7G), but LGKO mice exhibited an intermediate rate of glucose disposal after insulin stimulation (Figures 7F and 7H), which implies that GDF15, but not FGF21, is required for effective insulin action in mice fed an HFD. Because of the lower hepatic lipid accumulation in LKO mice fed an HFD, we next analyzed the liver histology of double knockout mice. LGKO livers contained larger numbers of intracellular lipid droplets than LKO mice, whereas LFKO livers contained fewer lipid droplets (Figure 7I). This suggests that the GDF15 that is secreted in response to the ISR^mt^ may protect against hepatic fat deposition in LKO mice fed an HFD.

## Discussion

Recent studies have revealed that mitochondrial OxPhos dysfunction or proteotoxic stress can elicit an adaptive hormetic response to overcome the initiating abnormalities, which are paradoxically associated with improvements in systemic energy metabolism (Bhaskaran et al., 2018; Kleinridders et al., 2013; Pospisilik et al., 2007) and greater longevity in multiple organisms (Feng et al., 2001; Houtkooper et al., 2013; Kirchman et al., 1999; Liu et al., 2005; Owusu-Ansah et al., 2013). These benefits are thought to be mediated by metabokines (Chung et al., 2017b; Durieux et al., 2011; Kim et al., 2013; Ost et al., 2016). The liver is one of the first organs to respond to dietary factors (Kraegen et al., 1991) and demonstrates metabolic plasticity and flexibility of its mitochondria to permit adaptation to changes in bioenergetic demand (Koliaki and Roden, 2016). Liver-specific *Cpt2*-deficient mice exhibited improvements in metabolism that were associated with high serum concentrations of GDF15 and FGF21 (Lee et al., 2017), but the direct effects of changes in the secretion of these hepatic metabokines on energy metabolism were not described. Moreover, almost all studies have displayed the concurrent induction of GDF15 and FGF21 in response to key regulators of ISR^mt^, including CHOP and ATF4, but did not distinguish the metabolic role of each metabokine. In the present study, we created a mouse model of liver-specific OxPhos dysfunction and characterized the hepatic adaptive response, which we found to involve both cell-autonomous and cell non-autonomous signaling. We suggest that mitochondrial stress in hepatocytes results in (1) insulin-independent alterations in cellular energy metabolism and (2) systemic modulation of energy homeostasis through the different actions of the hepatic metabokines GDF15 and FGF21.

In LKO mice, the reduction in hepatic OxPhos activates glycolysis and insulin-independent glucose uptake, which seems to compensate for the energy deprivation. Although the hepatocytes of LKO mice use different pathways for their energy production, the size of glycogen storage granules and the expression of genes involved in gluconeogenesis and lipogenesis were lower in LKO livers. Although the detailed mechanism underlying the reduction in gluconeogenesis associated with OxPhos dysfunction was not defined in the present study, we identified reductions in the expression of transcription factors that regulate glucose and lipid metabolism, including *Foxo3*, *ChREBP,* and *Srebp1/2*, in the livers of LKO mice, which suggests that there is cross talk between mitochondrial OxPhos and the nucleus to maintain gluconeogenesis and lipid metabolism. Another effect of mitochondrial OxPhos deficiency was that hepatic insulin signaling, as assessed by the measurement of insulin-induced AKT phosphorylation, was markedly impaired in LKO livers. In contrast to the phenotype of the LKO mice, liver-specific apoptosis-inducing factor (*Aif*) knockout mice exhibited an improvement in hepatic insulin signaling, even though hepatic OxPhos was also impaired (Pospisilik et al., 2007). This discrepancy may be due to the differing levels of proteotoxic or oxidative stress. A deficiency of hepatic *Aif* does not change the intracellular or extracellular reactive oxygen species (ROS) concentrations compared with controls, but *Crif1*-deficient cells exhibit significantly higher mitochondrial ROS production (Kim et al., 2012) and proteotoxic stress, which lead to additional impairments in mitochondrial OxPhos.

In the present study, we have demonstrated that GDF15 and FGF21 are hepatic metabokines that modulate systemic energy metabolism in LKO mice (Figure 7J). Metabokines are well-known biomarkers of mitochondrial pathology (Davis et al., 2016), as well as important regulators of systemic energy metabolism. The promoters of *Gdf15* and *Fgf21* contain binding sites for transcription factors, such as CHOP and ATF4, respectively (Chung et al., 2017b; De Sousa-Coelho et al., 2012), which increase the levels of both metabokines during the ISR^mt^. Most studies conducted to date focused on the role of single metabokines in the regulation of insulin sensitivity and EE, whereas we have distinguished the differential effects of hepatic metabokines using double knockout LKO/*Gdf15*^*−/−*^ (LGKO) and LKO/*Fgf21*^*−/−*^ (LFKO) mice. We have identified a role for GDF15 in the regulation of body mass, fat mass, and insulin-dependent glucose disposal in LKO mice, whereas FGF21 improves insulin sensitivity and glucose homeostasis. Moreover, FGF21 ablation reduced EE and the induction of UCP1 in the iWAT of chow diet-fed LKO mice. Interestingly, we also found that GDF15 protects against hepatic steatosis and insulin resistance in mice fed an HFD, which was in contrast to our finding of fewer hepatic lipid droplets in FGF21-deficient mice. Although the underlying mechanism whereby GDF15 protects against hepatic steatosis is unclear, our data suggest that GDF15 and FGF21 have differing roles in liver fat homeostasis.

Recent studies have demonstrated effects of FGF21 (Ost et al., 2016) and GDF15 (Ost et al., 2020) secreted by muscle using skeletal muscle-specific *Ucp1* transgenic (TG) mice. The TG mice exhibited a skeletal muscle-specific mitochondrial stress response, but an improvement in systemic energy metabolism. However, contrary to our findings, these studies demonstrated that skeletal muscle-derived FGF21 was not required for the improvements in EE and insulin sensitivity (Ost et al., 2016). This discrepancy may be explained by differences in the responsiveness of mice of different ages to FGF21. The previous study was conducted in 40-week-old mice, and the serum FGF21 concentrations of the wild-type mice were ∼2 ng/mL, which was 10 times higher than the concentrations in the control mice in the present study, which suggests that older mice may be FGF21 resistant. Although the molecular mechanism underlying FGF21 resistance requires elucidation (Markan et al., 2017), aging is known to increase the FGF21 concentration and is negatively associated with FGF21 responsiveness (Villarroya et al., 2018). Another difference between the present study and that of the *Ucp1*-TG mice regards whether GDF15 plays an important role in the UCP1-dependent browning of iWAT. Browning in the *Ucp1*-TG mice was abolished in TG/*Gdf15*^*−/−*^ mice, which contrasts with our finding that genetic ablation of *Gdf15* does not alter *Ucp1* induction in LKO mice. GDF15 has an anti-inflammatory role in adipose-resident macrophages because it promotes M2-like polarization (Jung et al., 2018), which is favorable for browning (Nguyen et al., 2011). Given that adipose inflammation gradually increases with age (Schaum et al., 2019), the potential for browning would be maximized in older mice through the activation of M2 macrophages. Our *in vivo* experiments were conducted in young mice (8–10 weeks of age), which may have reduced the impact of GDF15 on the immune cells that favor the browning of adipose tissue.

In our previous studies, we developed mice with mitochondrial OxPhos dysfunction caused by *Crif1* deficiency in a tissue-specific manner (brain, adipose tissue, skeletal muscle, β-cell, and macrophage) (Choi et al., 2020; Chung et al., 2017b; Jung et al., 2018; Kim et al., 2012, 2015). However, induction of metabokines (GDF15, FGF21) were not observed in every genetic model. Mice with *Crif1* deficiency in β-cells and macrophages exhibited normal or reduced expression of metabokines, respectively, whereas deletion of *Crif1* in adipose and skeletal muscle showed significantly increased metabokine levels, suggesting that the mitohormetic effects and induction mechanism can vary tissue specifically.

As mentioned, we developed adipose-specific *Crif1* KO mice (AdKO) (Choi et al., 2020) and observed the roles of GDF15 and FGF21 in this model. We postulated that the metabolic effects of these metabokines can vary depending on which tissue produces the metabokines. Several studies suggest that liver, but not the adipose tissue, produces the circulating FGF21 in DIO mice (Markan et al., 2014) or in mice exposed to cold (Ameka et al., 2019), which regulated glucose disposal or core body temperature, respectively. At least in these conditions (DIO, acute cold exposure), FGF21 derived from adipose tissue was insufficient to enhance the glucose disposal or regulate core body temperature. In agreement with these studies, AdKO mice fed a chow diet showed only modest changes in glucose and insulin tolerance as well as EE despite induction of GDF15 and FGF21 in white adipose tissue. In contrast to ADKO mice, LKO mice fed a chow diet showed improved insulin tolerance. The effects on hepatic steatosis in mice fed an HFD also differed between the two studies. In DIO-AdKO mice, both GDF15 and FGF21 protected from hepatic fat accumulation, whereas in DIO-LKO mice, only GDF15 showed a protective effect on hepatic fat accumulation.

The previous study using male GDF15KO mice exhibited similar EE and cumulated food intake as controls, but the fat mass was significantly increased in GDF15KO mice at 12–14 weeks of age (Tsai et al., 2013). However, the male GKO mice used in this study manifested similar body and fat mass with controls at 10 weeks of age, which was consistent with another study observing the body and fat mass in GDF15KO mice up to 95 weeks of age (Ost et al., 2020). Moreover, unlike with the previous study, our male GKO and LGKO mice exhibited significantly increased cumulative food intake for 5 days. This discrepancy can be caused by differences in the normalization of food intake. The previous study displayed modest changes in food intake, which was normalized to the total body weight, despite a different body composition (Tsai et al., 2013). However, according to the guidelines for measuring energy intake and expenditure in mice, simple division by body weight can overlook the relationship between body composition (fat and lean mass) and energy metabolism (Tschop et al., 2011). This approach can only be adjusted in the case that the intercept of regression line between mass and EE is zero.

GDF15 has a potent anorexigenic effect that is exerted via activation of the brainstem receptor GFRAL (Hsu et al., 2017; Yang et al., 2017). Recent work has suggested that GDF15 administration affects food intake by activating GFRAL (Borner et al., 2020). Although the mice were administered a low dose of GDF15 (20 μg/mL) in this study, the dose is still high compared with the normal physiological levels of GDF15 (∼100 pg/mL in C57BL/6 mice) (Patel et al., 2019). However, LKO mice with serum GDF15 concentrations of ∼300 pg/mL did not exhibit any differences in food intake, which was consistent with the phenotypes of several mouse models that had endogenous serum GDF15 concentrations of ∼400 pg/mL (Choi et al., 2020; Kim et al., 2018; Ost et al., 2020). Pharmacological doses of recombinant GDF15 induce emesis and nausea, and lead to about 60-fold higher concentrations than the serum GDF15 concentrations in the LKO mice. Further evidence may be obtained from study of the soluble form of GFRAL, GFRAL-B (Li et al., 2005). GFRAL has two splice variants: GFRAL-A, a membrane-bound full-length form, and GFRAL-B, which lacks the cysteine-rich domain 3. Although the expression of GFRAL-A and B is restricted to certain brain regions, it still needs to be determined whether the soluble form of GFRAL can bind to circulating GDF15. GDF15 activity and the concentration threshold required for its effects on systemic energy metabolism may be regulated by this soluble form of GFRAL. Therefore, further study is required to determine whether soluble GFRAL has a role in metabolism.

### Limitations of the study

Although the expression of *Fgf21* and *Gdf15* was only increased in the livers of LKO mice, it is difficult to exclude an effect of the basal expression of these genes in other tissues on the mouse phenotype. According to previous study findings, global *Gdf15* knockout mice does not alter blood glucose, insulin tolerance, or EE in male mice (Tsai et al., 2013). In addition, *Fgf21* knockout mice did not show a difference in blood glucose in the fed state (Liang et al., 2014; Potthoff et al., 2009). However, during fasting conditions, *Fgf21* deficiency markedly impaired expression of key enzymes for gluconeogenesis (Liang et al., 2014). Furthermore, we only evaluated WAT browning by FGF21 and GDF15 *in vivo* and hence cannot exclude indirect effects of these metabokines on metabolism. Finally, in a recent study, GDF15 was shown to be required for the effect of the hepatic sympathetic outflow on triglyceride metabolism (Luan et al., 2019); therefore, the hepatic abnormalities in the LKO mice may have altered some of the central actions of endogenous GDF15.

### Resource availability

#### Lead contact

Further information and requests for resources and reagents should be directed to and will be fulfilled by Minho Shong (Lead Contact and Corresponding Author; minhos@cnu.ac.kr) or Hyon-Seung Yi (Corresponding Author; jmpbooks@cnu.ac.kr).

#### Materials availability

This study did not generate new unique reagents.

#### Data and code availability

The RNA sequencing data for the liver, iWAT, GM, and hypothalamus generated during this study have been deposited in the Gene Expression Omnibus (GEO). They are available with accession number GSE149553.

## Methods

All methods can be found in the accompanying Transparent Methods supplemental file.

## Supplemental information

Document S1. Transparent methods, Figures S1–S6, and Tables S1 and S4

Table S2. DEG lists, related to Figure 4 and S4

Table S3. Functional annotation, related to Figure 4 and S4
