# Supplementary material for: Differential roles of GDF15 and FGF21 in systemic metabolic adaptation to the mitochondrial integrated stress response
Source: iScience. 2021 Feb 12;24(3):102181. doi: 10.1016/j.isci.2021.102181 (PMC7920832; doi:10.1016/j.isci.2021.102181)
Supplement: Document S1. Transparent methods, Figures S1–S6, and Tables S1 and S4 [file mmc1.pdf]

iScience, Volume *24*

Supplemental information

Differential roles of GDF15 and FGF21 in systemic metabolic adaptation to the mitochondrial integrated stress response

Seul Gi Kang, Min Jeong Choi, Saet-Byel Jung, Hyo Kyun Chung, Joon Young Chang, Jung Tae Kim, Yea Eun Kang, Ju Hee Lee, Hyun Jung Hong, Sang Mi Jun, Hyun-Joo Ro, Jae Myoung Suh, Hail Kim, Johan Auwerx, Hyon-Seung Yi, and Minho Shong

Transparent Methods

Mice

All the experiments were performed in homozygous male mice. Liver-specific *Crif1*-deleted mice (LKO) were generated by crossing Albumin-Cre transgenic mice and floxed-*Crif1* mice (*Crif1*$^{f/f}$, control). Albumin-Cre mice were purchased from the Jackson Laboratory (Tg[Alb-Cre]21Mgn) and had been backcrossed on to the C57BL/6J background, and floxed-*Crif1* mice were generated as previously described (Kwon et al., 2008). GDF15 null mice (*Gdf15*$^{-/-}$, GKO) and FGF21 null mice (*Fgf21*$^{-/-}$, FKO) were kindly provided by Dr. S. Lee (Johns Hopkins University School of Medicine, Baltimore, MD) and Dr. N. Itoh (Kyoto University Graduate School of Pharmaceutical Sciences), respectively. These null mice were crossed with floxed-*Crif1* and Albumin-Cre transgenic mice to generate LGKO (LKO/*Gdf15*$^{-/-}$) and LFKO (LKO/*Fgf21*$^{-/-}$), respectively. The mice were housed in a specific pathogen-free facility at the Preclinical Research Centre of Chungnam National University Hospital (CNUH-019-A0077) under a 12 h light/12 h dark cycle, at an ambient temperature of 22±2 °C and at a relative humidity of 40–60%. They were fed a chow diet (Teklad global 18% protein, 2918C, ENVIGO) and used in the experiments at 8–10 weeks of age. Diet-induced obesity (DIO) was induced by feeding an HFD (60% of total energy intake as fat, TD.06414, ENVIGO) for 8 weeks, starting at 6 weeks of age. All the experimental procedures complied with the guidelines of and were approved by the Institutional Animal Care and Use Committees.

Primary hepatocyte isolation and culture

As previously described (Kang et al., 2017; Yi et al., 2014), *in situ* perfusion was conducted in control and LKO mice for the isolation of primary hepatocytes using EGTA solution (0.5 mM EGTA, 25 mM Tricine, 5.4 mM KCl, 0.44 mM KH_2PO_4, 140 mM NaCl, 0.34 mM Na_2HPO_4, pH 7.2) and collagenase solution (0.8 mg/ml collagenase type I in Hank's Balanced Salt Solution; Worthington, Freehold, NJ, USA) for 30 min. The suspensions were then filtered using 70 μm cell strainers (BD Falcon, Millville, NJ, USA) and centrifuged at 1,000 × *g* for 5 min. The pelleted cells were resuspended and isolated with 40% Percoll solution (GE Healthcare, Buckingham, UK) at 1,200 × *g* for 10 min at 4°C, and the isolated primary hepatocytes were seeded in Medium 199 (Sigma

Aldrich, MO, USA) containing 10% foetal bovine serum (Thermo Fisher Scientific, Waltham, MA, USA) and 1% penicillin and streptomycin (Welgene, Daegu, South Korea) at a density of 3×10^5 cells per well.

Blue native polyacrylamide gel electrophoresis (BN-PAGE)

For the mitochondrial isolation, 30 mg of liver tissue were homogenized in isolation buffer (210 nM mannitol, 70 mM sucrose, 1 mM EGTA, 5 mM HEPES, pH 7.2) using WiseStir (HS-30E, Daihan, Wonju, South Korea). The samples were then centrifuged at 600 × *g* for 10 min at 4°C and the supernatants were re-centrifuged at 17,000 × *g* for 10 min at 4°C. The pelleted mitochondria were resuspended and the constituent proteins separated using a Native PAGE Novex Bis-Tris Gel system (Invitrogen). To quantify the mitochondrial OxPhos complexes, 30 μg of each mitochondrial fraction in Native PAGE sample buffer (Invitrogen) and 10% n-dodecyl-β-D-maltoside were loaded onto Native PAGE 3–12% Bis-Tris gels. After electrophoresis, the separated proteins were transferred to PVDF membranes using iBlot gel transfer stacks (Invitrogen) and fixed in 8% (v/v) acetic acid. After overnight drying, the membranes were incubated with anti-OxPhos complex cocktail (#457999, Invitrogen; sc-58347, Santa Cruz) for 90 min and visualized using a Western Breeze Chromogenic Western Blot Immuno-detection kit (Invitrogen).

Oxygen consumption rate

The mitochondrial OCR and extracellular acidification rate (ECAR) were measured using a Seahorse XF-24 extracellular flux analyzer (Seahorse Bioscience, North Billerica, MA, USA). Primary hepatocytes were isolated and cultured overnight at a concentration of 1×10^4 cells per well on Seahorse XF-24 plates. The calibration cartridge (#102416-100, Seahorse Bioscience) was hydrated using calibration buffer (#100840-000, Seahorse Bioscience) 1 day prior to the analysis and incubated at 37°C in a non-CO_2-containing incubator. The cells were then washed and incubated in XF Assay medium lacking sodium bicarbonate (#102365-100, Seahorse Bioscience) at 37°C in a non-CO_2-containing incubator for 1 h. The calibration plate was then loaded into the Seahorse XF-24 analyzer and calibration was performed for 30 min at 37°C. The OCR and ECAR were then analyzed at baseline and following the addition of three mitochondrial inhibitors: oligomycin (2 μg/ml), CCCP

(10 μM) and rotenone (1 μM). The measurements were programmed for four cycles of 2 min of mixing, 2 min of waiting and 3 min of measurement at both baseline and following the addition of each inhibitor.

Glycolysis assay

Glycolysis was assessed in primary hepatocytes by measuring the ECAR using a Seahorse XF Glycolysis Stress Test Kit (#103020-100, Seahorse Bioscience) and the Seahorse XF-24 extracellular flux analyzer. Primary hepatocytes were seeded at a concentration of 1×10^4 cells per well in Seahorse XF Culture Microplates. The calibration cartridge was hydrated at 37°C in a non-CO_2-containing incubator 1 day before the assay. The growth medium was replaced with Seahorse XF Base Medium (#103193-100, Seahorse Bioscience) containing glutamine (1 mM) and the cells were incubated at 37°C in a non-CO_2-containing incubator for 1 hour. To measure the ECAR, three reagents were used: glucose (10 mM), oligomycin (1 μM) and 2-deoxyglucose (50 mM). These measurements were conducted over three cycles of 2 min of mixing, 2 min of waiting and 3 min of measurement at baseline and following the addition of each chemical.

Transmission electron microscopy

The livers of control and LKO mice were cut into 1 mm^3 specimens and pre-fixed with 2.5% glutaraldehyde in pH 7.4 phosphate buffer (0.1 M) overnight at 4°C. The specimens were then washed in phosphate buffer three times for 10 min, which was followed by post-fixation using 1% osmium tetroxide (0.1 M) in phosphate buffer for 1 h at 4°C. After dehydration in a graded series of ethanol mixtures (50%, 75%, 90%, 95% and 100%), the ethanol were replaced by propylene oxide and mixtures of Embed 912 resin (EMS). After embedding and polymerization in fresh resin for 24 hours, the blocks were sectioned at 80 nm on an ultramicrotome (Leica, Bensheim) using a diamond knife and mounted on 200-mesh copper grids. The sections were then stained with uranyl acetate and lead citrate, and examined using a Leo912 transmission electron microscope (Carl Zeiss, Oberkochen) at 120 kV.

Quantitative real-time PCR (QPCR)

Tissues were homogenized using a TissueLyser II (Qiagen) and RNA was isolated using TRIzol™ Reagent (15596018, Life Technologies, Thermo Fisher Scientific). cDNA was synthesized from the RNA (5 μg) using Oligo(dT)15 Primer (Promega) and M-MLV Reverse Transcriptase (Thermo Fisher Scientific). QPCR was performed using a 7500 Fast Real-Time PCR System (Applied Biosystems, Carlsbad, CA) and GoTaq qPCR Master Mix (BRYT Green, Promega). Relative quantification was performed using Applied Biosystems 7500 Software (ver. 2.0.6) and the ΔΔCT method, and gene expression was normalized to that of 18s rRNA. The primers used are listed in Table S1.

Glucose uptake assay

Isolated primary hepatocytes were seeded at a density of 1×10^5 cells per well in a sterile 96-well plate and incubated for 24 h before the assay. The cells were serum-starved for 2 h and incubated in 2% BSA/KPRH buffer (20 mM HEPES, 5 mM KH_2PO_4, 1 mM $MgSO_4$, 1 mM $CaCl_2$, 136 mM NaCl, 4.7 mM KCl, pH 7.4) for 40 min. Some of the wells were treated with insulin (1 μM, I9278, Sigma Aldrich) in KPRH buffer for 20 min. Glucose uptake was measured using 2-DG and a Glucose uptake assay kit (ab 136955, Abcam, Cambridge, UK), according to the manufacturer's instructions. Glucose uptake was calculated according to the colorimetric change, which was measured using a microplate reader (VersaMax, Molecular Devices) at 412 nM at 2 min intervals.

Western blotting

Mouse tissues were homogenized using a TissueLyser II in lysis buffer (50 mM Tris-HCl, pH 7.4; 150 mM NaCl; 1 mM EDTA, pH 8.0; 0.1% Triton X-100) containing a protease inhibitor cocktail (#11836145001, Roche, Basel, Switzerland) and phosphatase inhibitors (04906837001, Roche) on ice for 30 min. After centrifugation at 16,000 g for 15 min, the protein concentrations of the supernatants were measured using a BCA protein assay (#23227, Thermo Fisher Scientific). Fifty micrograms of protein per sample were loaded onto 8–12% polyacrylamide gels and electrophoresis was performed. The separated proteins were then electrotransferred to 0.45 μm PVDF membranes (#IPVH00010, Millipore) at 200 mA for 2 h. Membranes were blocked with 5% skimmed milk (#T145.2, Roth) in TBS/T buffer (20 mM Tris, 150 mM NaCl, 0.1% Tween 20, pH 7.6) for 1 h and

then incubated with primary antibodies overnight at 4°C. After washing three times with TBS/T, the membranes were incubated with secondary antibodies for 1 h at room temperature and then visualized using ECL solution (#34580, Thermo Fisher Scientific). Target protein levels were normalized to those of β-actin, α-tubulin or glyceraldehyde 3-phosphate. The antibodies used are listed in Table S4.

Hematoxylin and eosin (H&E) staining

Liver, iWAT and BAT samples were obtained from male control, LKO, GKO, LGKO, FKO and LFKO mice that had been fed a chow or HFD (60% fat) for 8 weeks. The tissues were fixed in 10% neutral formalin (BBC Biochemical, Mt. Vernon, WA, USA) overnight and then embedded in paraffin wax. After sectioning (4 μm thickness), the tissue samples were deparaffinized by three immersions in xylene for 4 min each and washed with a graded ethanol series for 2 min each. After washing with distilled water for 4 min, the tissue sections were stained with hematoxylin (S2-5, YD Diagnostics, Yongin, South Korea) for 4 min and eosin (#HT1101128, Sigma Aldrich) for 3 min. The sections were then dehydrated with a graded ethanol series for 2 min each and immersed three times in xylene for 4 min each, and then mounted and examined using an upright microscope (#BX53, Olympus, Tokyo, Japan).

Glucose tolerance and insulin tolerance testing

For glucose (GTT) and insulin (ITT) tolerance testing, mice were fasted for 6 h from 08:00 to 14:00. GTT was conducted by the intraperitoneal injection of 2 g/kg glucose (dextrose, Choongwae) in chow diet-fed mice or 1 g/kg glucose in HFD-fed mice. For ITT, mice were injected intraperitoneally with 0.75 U/kg insulin (Humalog, Lilly, Indianapolis, IN, USA). After each injection, the glucose concentrations of tail vein blood were measured after 0, 15, 30, 60, 90 and 120 min using a glucometer (Accu-Chek Active, Roche).

Serum biochemical measurements

Blood was collected from a retro-orbital sinus, incubated at room temperature for 2 h and then centrifuged at 600 g for 5 min to obtain serum. Serum triglyceride, cholesterol, GOT/AST and GPT/ALT (#1650, #1450,

#3150, #3250, Fujifilm) were measured using a Fuji DRI-CHEM 7000i (Fujifilm, Tokyo, Japan). The insulin (80-INSMSU-E01, Alpco), GDF15 (MGD150, R&D Systems, Minneapolis, MN, USA), FGF21 (MF2100, R&D Systems), leptin (MOB00, R&D Systems), lactate (#K607-100, Bio Vision, Milpitas, CA, USA) and β-hydroxybutyrate (Free Style Optium H β-Ketone, Abbott, Illinois, USA) concentrations were determined in serum.

Body composition and indirect calorimetry

Lean body mass (LBM) and fat mass (FM) were measured by dual energy X-ray absorptiometry (DXA) (Medikors, Seongnam, South Korea) in control, LKO, GKO, LGKO, FKO and LFKO mice fed a chow diet or an HFD (60% fat). The total body, lean and fat masses were automatically quantified using InAlyzer (ver. 1.00). EE was measured by indirect calorimetry. Before this measurement, the mice were placed in individual metabolic cages and acclimatized for 2 days. Oxygen consumption (VO_2), carbon dioxide production (VCO_2), EE, respiratory exchange rate (RER), physical activity and food intake were measured every 3 minutes using an OxyletProTM system (Panlab, Barcelona, Spain). The mean values for the daytime (from 08:00 to 18:00) and night-time (from 18:00 to 08:00) were calculated using METABOLISM software (ver. 2.2, Panlab).

RNA sequencing

RNA was isolated from the liver, iWAT, GM and hypothalamus of 8-week-old control and LKO mice. To construct cDNA libraries, 1 μg RNA and a TruSeq RNA Library Prep Kit v2 (RS-122-2001, Illumina, San Diego, CA, USA) were used, and the results were quantified by QPCR using an 2100 Bioanalyzer (Agilent Technology Inc., Santa Clara, CA, USA). The libraries were used for 100 nt paired-end sequencing by an Illumina HiSeq4000 (Illumina). After removing the low-quality and adapter sequences using Trimmomatic, the reads were aligned with the *Mus musculus* genome (mm10) using HISAT (ver. 2.0.5) (Kim et al., 2015). Two types of indexes were used for alignment (a global, whole-genome index and tens of thousands of small local indexes), which were downloaded from the UCSC table browser (http://genome.uscs.edu). StringTie (ver. 1.3.3b) was used to assemble the transcript, and provided the relative abundance estimates as fragments per kilobase of exon per million fragments mapped (FPKM) values of the transcript or gene. After excluding the genes with

one more than zero FPKM values, the signal value (FPKM+1) was transformed to a base 2 logarithm and normalized by quantile normalization methods to reduce the systematic bias. These values were used for the analysis of the differential expressed genes (DEGs) in the mouse groups. The statistical significance of the DEG values was calculated using independent *t*-tests ($p<0.05$) and fold change ($|FC|\geq 2$). The false discovery rate (FDR), which estimates the frequency of type I statistical errors, was determined by adjusting the *p*-value using the Benjamini and Hochberg algorithm.

Bioinformatic analysis

Transcriptome data for the liver, iWAT and GM were used for bioinformatic analysis. The Database for Annotation, Visualization and Integrated Discovery (DAVID ver. 6.8, https://david.ncifcrf.gov) (Huang da et al., 2009) was used to functionally annotate the data. The transcripts that satisfied the $|FC|\geq 2$ and $p<0.05$ criteria were matched with Entrez IDs to identify up- and downregulated genes. GOTERM_BP, which is based on Gene Ontology, and KEGG_PATHWAYS (Kyoto Encyclopaedia of Genes and Genomes) were used as the annotation categories. Gene Set Enrichment Analysis (GSEA) was performed in the liver transcriptome using GSEA v4.1.0, as described previously (Mootha et al., 2003; Subramanian et al., 2005). Gene sets including C2 KEGG subset (186 gene sets) and C2 REACTOME subset (1532 gene sets) of canonical pathways were downloaded from the Molecular Signatures Database (MSigDB, http://software.broadinstitute.org/gsea). For the graphical analysis of the transcriptome, PermutMatrix (ver. 1.9.3) and Network2Canvas were used, as previously described (Caraux and Pinloche, 2005; Tan et al., 2013). For hierarchical clustering and the identification of network nodes, the normalized z-score, based on the FPKM of the transcriptome, was used and is visualized using a gradation of colors. Proteins secreted by the liver were classified using the PANTHER classification system (v.14.0) (Mi et al., 2013).

Quantification and statistical analysis

Statistical analyses were performed using IBM SPSS Statistics software (ver. 24, IBM, Armonk, NY, USA). Data are presented as means ± standard errors of the mean (SEMs). All the experimental data were analyzed using Student’s two-tailed *t*-test, one-way ANOVA followed by Scheff’s *post-hoc* test or analysis of covariance

(ANCOVA) with the covariate of total body mass. $P < 0.05$ was considered to represent statistical significance. The number of biological replicates (n), the number of independent experiments and the method of statistical analysis used are reported in the corresponding figure legends.

Supplemental Information

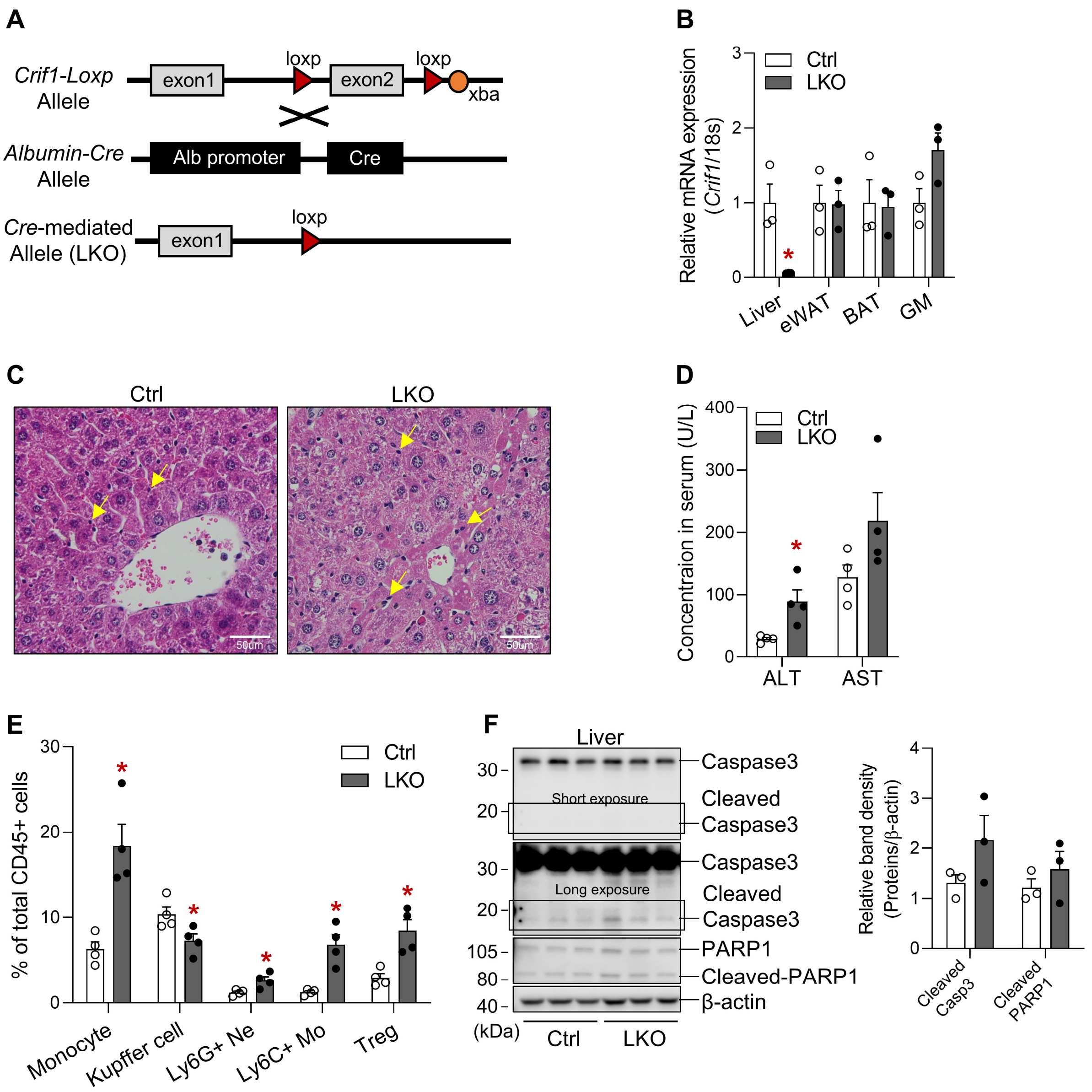

Figure S1. Liver-specific *Crif1*-deficient mice exhibit infiltration of mononuclear cells in the liver (Related to Figure 1).

(A) Schematic illustration of the deletion of exon2 of the *Crif1* allele on chromosome 8 through the crossing of *Crif1*$^{loxp/loxp}$ mice and Albumin-Cre transgenic mice.

(B) Quantitative PCR analysis of Crif1 mRNA expression in the liver, eWAT, BAT and gastrocnemius muscle (GM) of control (Ctrl) and LKO mice (n=3 biological replicates from two independent experiments).

(C) Representative haematoxylin and eosin-stained liver sections from Ctrl and LKO mice at 10 weeks of age

(n=4 biological replicates from two independent experiments). The yellow arrows indicate mononuclear cells in the liver.

(D) Serum ALT and AST activities in Ctrl and LKO mice at 11 weeks of age (n = 4 biological replicates from three independent experiments).

(E) FACS analysis of infiltrated monocytes (IM, $F4/80^{low}CD11b^{high}$), kupffer cells (KCs, $F4/80^{high}CD11b^{low}$), neutrophils ($CD11b^{high}Ly6G^{high}$), inflammatory monocyte ($CD11b^{high}LyC^{high}$), and Treg cells ($CD4^{high}CD44^{high}CD62L^{high}$) in liver of control and LKO mice at 8 weeks of age (n=4 per group).

(F) Western blot analysis of apoptotic markers in the livers of Ctrl and LKO mice at 10 weeks of age (n=3 biological replicates, left). The results of one representative experiment of the two performed is shown. The relative band densities of cleaved caspase 3 and PARP1 in the liver (right). There were no significant differences. The mice were fed a chow diet (6% fat). Data are mean ± SEM and were analysed using Student's *t*-test (**p*<0.05 *vs.* Ctrl).

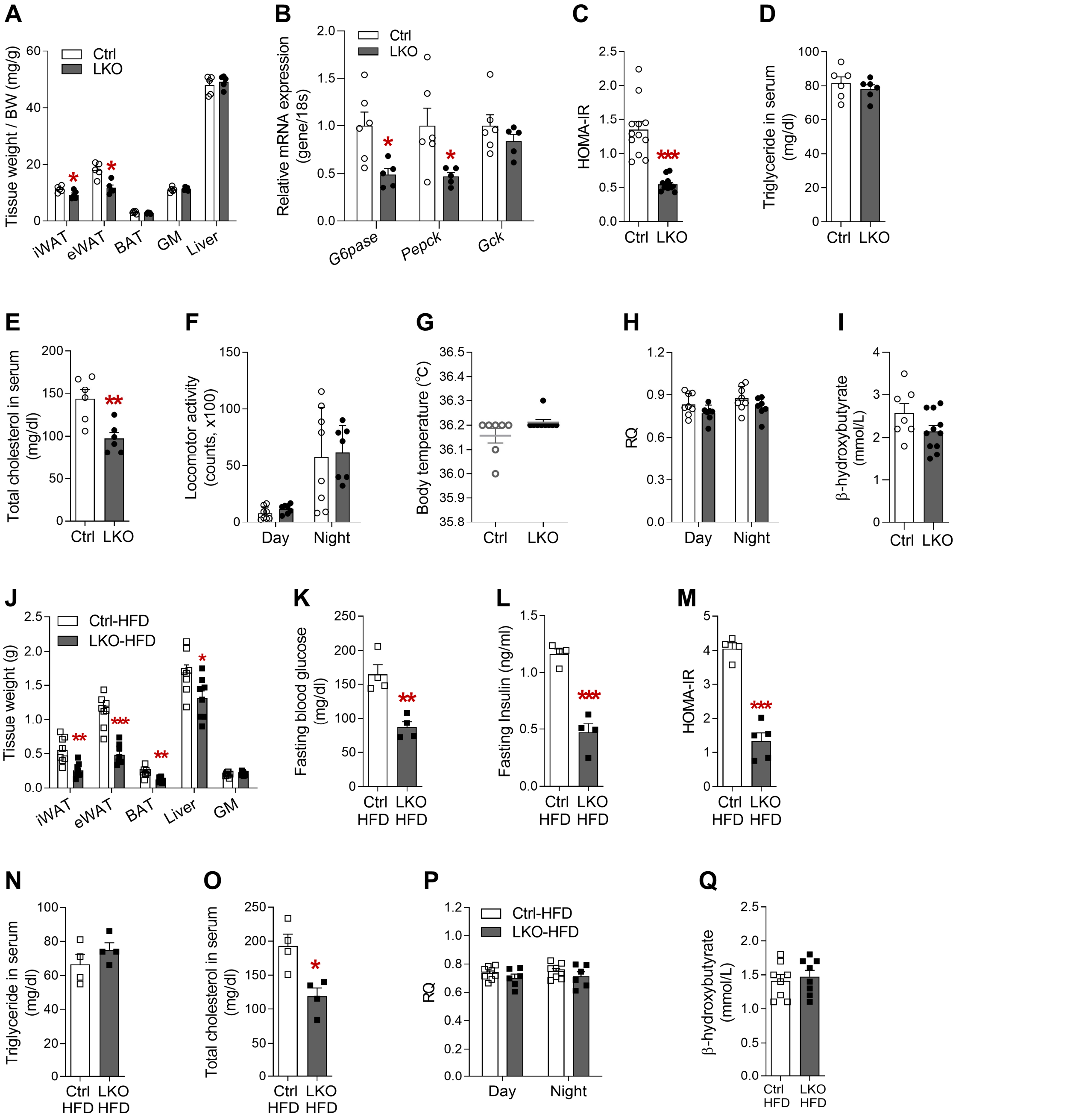

Figure S2. Liver-specific *Crif1*-deficient mice have low fat mass and high insulin sensitivity when consuming a chow diet and high-fat diet (Related to Figure 2).

Mice fed a chow diet were used in (A-I) and mice fed an HFD for 8 weeks were used in (J-Q).

(A) Tissue mass per unit body mass in Ctrl and LKO mice (n=5 biological replicates from three independent experiments).

(B) Quantitative PCR analysis of the expression of genes involved in gluconeogenesis in Ctrl and LKO livers (n=5–6 biological replicates from two independent experiments). Mice were fasted for 6 hours.

(C) HOMA-IR, calculated using the serum insulin and glucose concentrations of Ctrl and LKO mice (n=12 biological replicates).

(D and E) Serum triglyceride (D) and total cholesterol (E) in Ctrl and LKO mice (n=6 biological replicates from two independent experiments).

(F) Physical activity, assessed using the locomotor activity count in Ctrl and LKO mice housed in individual cages (n=7–8 biological replicates).

(G) Body surface temperature, measured using an infrared thermometer, in Ctrl and LKO mice (n= 7 or 10 biological replicates from two independent experiments).

(H) RQ in mice fed a chow diet (n=7–8 biological replicates).

(I) Serum β-hydroxybutyrate in Ctrl and LKO mice that had been fasted for 16 hours (n=7 or 11 biological replicates).

(J) Tissue masses in Ctrl and LKO mice fed a high-fat diet (n=8 biological replicates from two independent experiments).

(K-M) Blood glucose (K), serum insulin (L) and HOMA-IR (M) in 6 h-fasted Ctrl and LKO mice fed a high-fat diet (n=4 biological replicates).

(N and O) Serum triglyceride (N) and total cholesterol (O) of Ctrl and LKO mice fed a high-fat diet (n=4 biological replicates).

(P) RQ of Ctrl and LKO mice, fed a high-fat diet, at 14 weeks of age (n= 6 or 8 biological replicates).

(Q) Serum β-hydroxybutyrate of Ctrl and LKO mice, fed a high-fat diet, at 14 weeks of age (n=8 biological replicates). Mice were fasted for 18 h before the measurement. Data are mean ± SEM and were analysed using Student's *t*-test (**p*<0.05, ***p*<0.01, ****p*<0.001 *vs.* Ctrl).

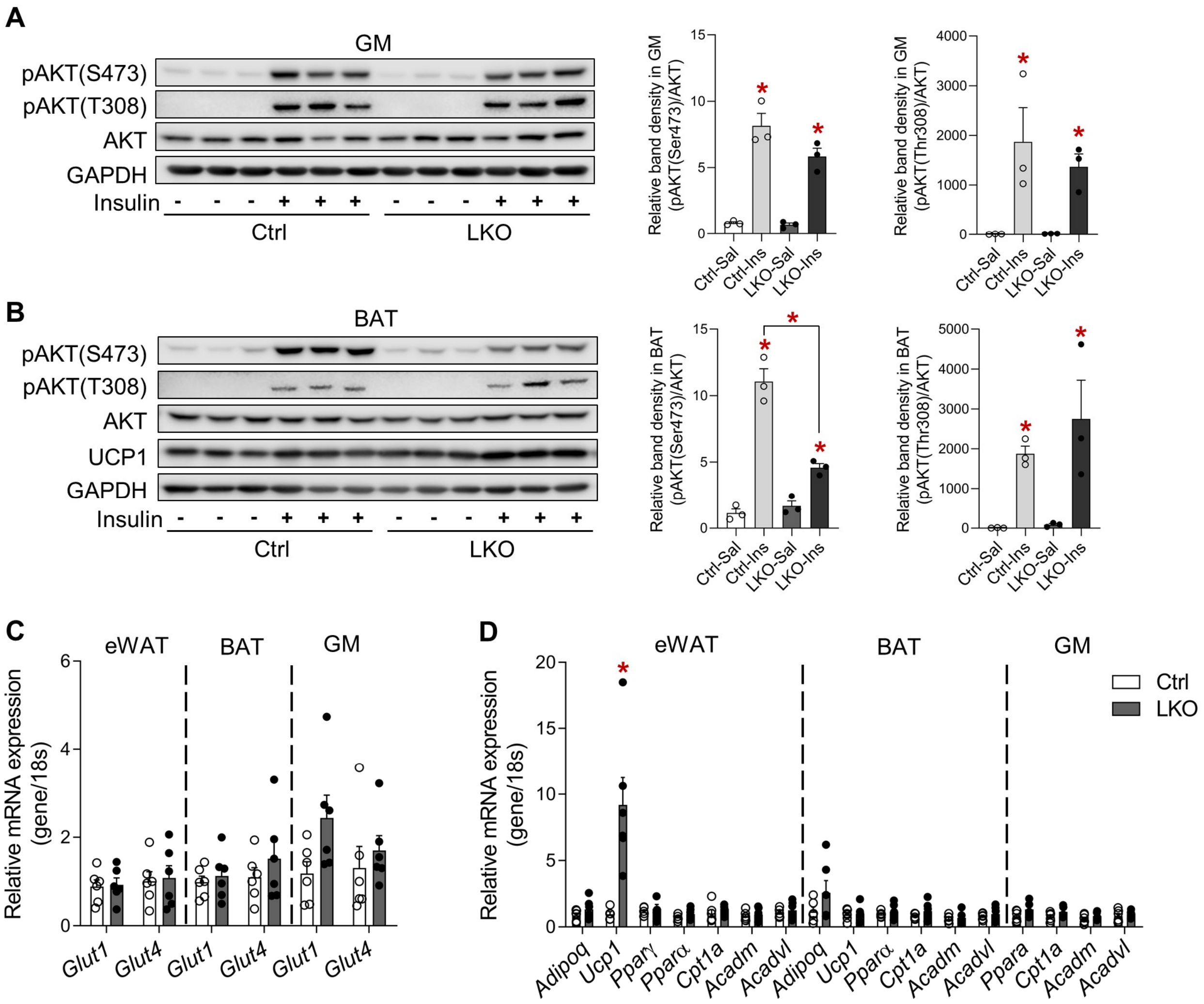

Figure S3. Liver-specific *Crif1*-deficient mice exhibit normal insulin signalling and normal mRNA expression of fatty acid metabolic genes in GM and BAT (Related to Figure 3).

(A and B) Western blot analysis of AKT phosphorylation in the GM (A) and BAT (B) of Ctrl and LKO mice at 9 weeks of age (n=3 biological replicates). Mice were fasted for 6 h and then insulin was administered intraperitoneally (4 U/kg).

(C) Quantitative PCR analysis of *Glut1* and *Glut4* mRNA expression in the eWAT, BAT and GM of Ctrl and LKO mice at 9 weeks of age (n=6 biological replicates from two independent experiments).

(D) Quantitative PCR analysis of fatty acid oxidation gene expression in the eWAT, BAT and GM from Ctrl and LKO mice at 9 weeks of age (n=5–6 biological replicates from two independent experiments). Data are expressed as the mean ± SEM and were analysed using two-way ANOVA followed by Scheff's *post-hoc* test in (A) and (B), and Student's *t*-test in (C) and (D) (*p<0.05 *vs.* Ctrl).

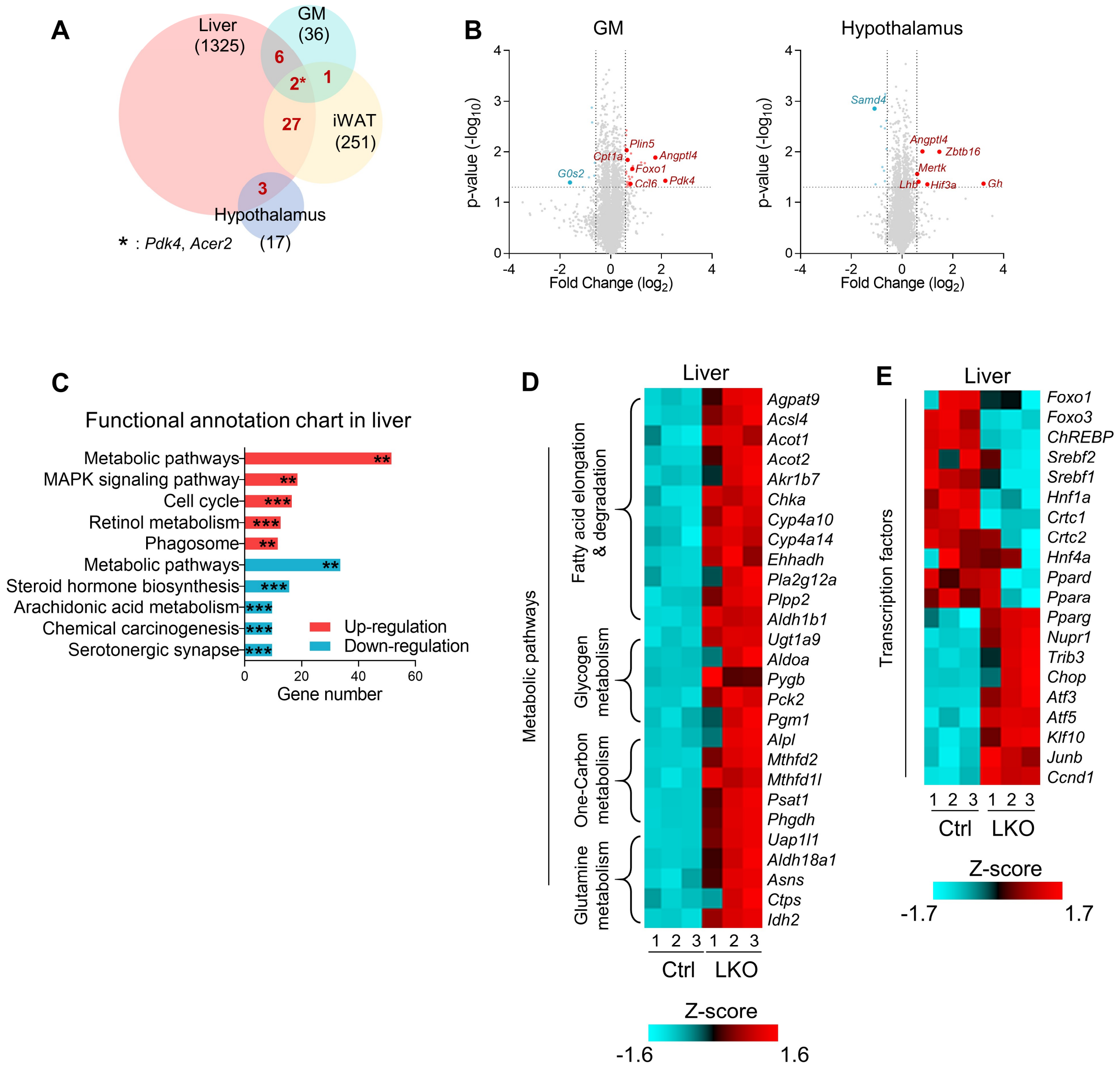

Figure S4. RNA sequencing suggests the downregulated lipid metabolism in the liver of LKO mice (Related to Figure 4).

(A) Venn diagram of the differentially expressed genes (DEGs) obtained from RNA sequencing in the liver, iWAT, GM and hypothalamus of mice (n=3 biological replicates). The numbers represent the differentially expressed transcripts in LKO mice, compared with Ctrl mice (fold difference ≥ ±1.5-fold, $p < 0.05$).

(B) Volcano plot showing the DEGs in GM (left panel) and hypothalamus (right panel) of LKO mice compared with controls. The colored dots indicate the DEGs with ≥ ±1.5-fold difference from Ctrl mice. The red and blue

dots indicate the upregulated and downregulated transcripts, respectively.

(C) Top-ranked functional annotation chart for the liver, using DEGs (DEGs; ≥ ±1.5-fold difference from Ctrl). Functional annotation was categorised using the KEGG pathway in DAVID (ver.6.8) and ordered according to gene number.

(D) Heat map showing the DEGs termed as ‘Upregulation-Metabolic pathways’ in (C).

(E) Heat map showing the differentially expressed transcription factors that regulate glucose and lipid metabolism (downregulation) and the cellular stress response (upregulation) in the livers of Ctrl and LKO mice (n=3 biological replicates). The data in (C) were analysed using a modified Fisher’s Exact *p*-value (*$p<0.05$, **$p<0.01$, ***$p<0.001$).

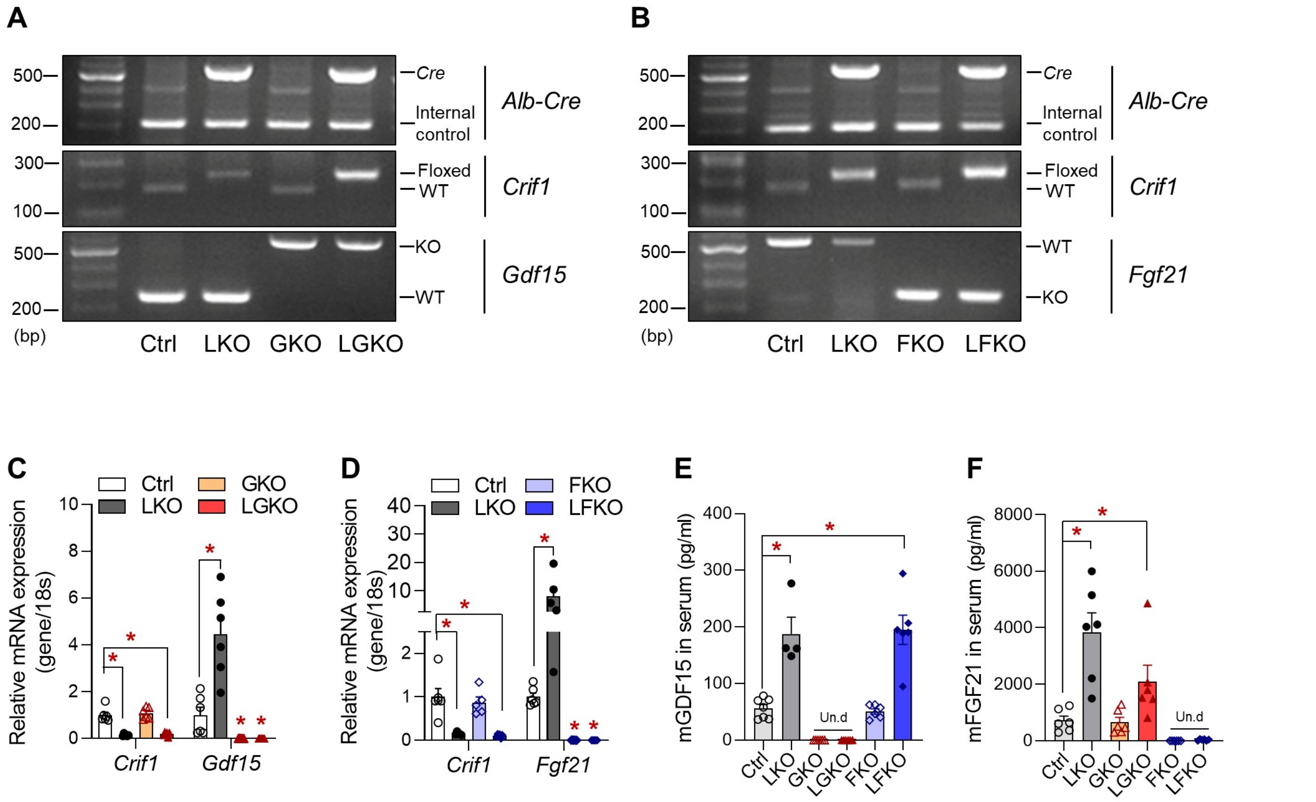

Figure S5. LGKO and LFKO mice were generated by crossing LKO and global GKO (*Gdf1*$^{-/-}$) and LKO and global FKO (*Fgf21*$^{-/-}$) mice, respectively (Related to Figure 5).

(A) PCR genotyping results for control mice (Ctrl, *Crif1*$^{f/f}$), liver-specific *Crif1*-null mice (LKO, *Crif1*$^{f/f,Alb}$), global *Gdf15*-null mice (GKO, *Gdf15*$^{-/-}$) and liver-specific *Crif1*- and global *Gdf15*-null mice (LGKO, *Crif1*$^{f/f,Alb}$/*Gdf15*$^{-/-}$). The PCR bands show the DNA amplification of *Cre* (565 bp), *Crif1* (*Crif1*-WT, 171 bp; *Crif1-loxp*, 205 bp) and *Gdf15* (*Gdf15*-WT, 228 bp; *Gdf15*-KO, 598 bp).

(B) PCR genotyping results for control mice (Ctrl, *Crif1*$^{f/f}$), liver-specific *Crif1*-null mice (LKO, *Crif1*$^{f/f,Alb}$), global *Fgf21*-null mice (FKO, *Fgf21*$^{-/-}$), and liver-specific *Crif1*- and global *Fgf21*-null mice (LFKO, *Crif1*$^{f/f, Alb}$/*Fgf21*$^{-/-}$). The PCR bands show the DNA amplification of *Cre* (565 bp), *Crif1* (*Crif1*-WT, 171 bp; *Crif1-loxp*, 205 bp) and *Fgf21* (*Fgf21*-WT, 540 bp; *Fgf21*-KO, 240 bp).

(C and D) Quantitative PCR analysis of *Crif1* and *Gdf15* mRNA expression (C), and *Crif1* and *Fgf21* mRNA expression (D) in the liver of mice (n=6 biological replicates).

(E and F) Serum GDF15 protein (E) and FGF21 protein (F) in Ctrl, LKO, GKO, LGKO, FKO and LFKO mice

(n=4–7 biological replicates from two independent experiments).

The mice were studied at 8–9 weeks of age and fed a chow diet. Data in (C-F) are mean ± SEM. Statistical analyses were performed using ANOVA followed by Scheff's post-hoc test in (C-F) (*$p<0.05$, **$p<0.01$, ***$p<0.001$ vs. Ctrl).

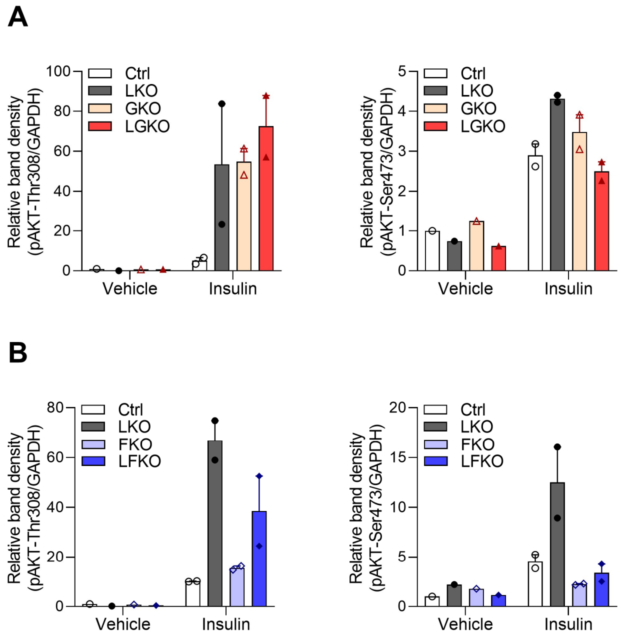

Figure S6. Genetic ablation of *Gdf15* and *Fgf21* in LKO mice attenuated the phosphorylation of AKT after insulin stimulation (Related to Figure 6).

(A) Relative band of phosphorylation of AKT at Thr308 (left panel) and Ser473 (right panel) in iWAT of Ctrl, LKO, GKO, LGKO mice fed a chow diet. Mice were fasted for 6 h and then administered intraperitoneally with insulin (4 U/kg).

(B) Relative band of phosphorylation of AKT at Thr308 (left panel) and Ser473 (right panel) in iWAT of Ctrl, LKO, FKO, LFKO mice fed a chow diet. Mice were fasted for 6 h and then administered intraperitoneally with insulin (4 U/kg). One representative experiment of the two conducted is shown. As less biological replicates within the group, statistical analysis were not conducted.

Table S1. Primers used for the quantitative PCR analysis of mRNA expression in mice

Gene	Forward (5′→3′)	Reverse (5′→3′)
Acadm	TGACGGAGCAGCCAATGA	TCGTCACCCTTCTTCTCTGCTT
Acadvl	TTACATGCTGAGTGCCAACATG	CGCCTCCGAGCAAAAGATT
Aco	ATGACCCCACTTCCTGACAC	GAAGGTCAGCCACCATGATT
Adipoq	TCTCCTGTTCCTCTTAATCCTGCC	CATCTCCTTTCTCTCCCTTCTCTCC
Atf3	TGCCTGCAGAAAGAGTCAGA	CCTTCAGCTCAGCATTCACA
Atf4	GGGTTCTGTCTTCCACTCCA	AAGCAGCAGAGTCAGGCTTTC
Atf5	TGGGCTGGCTCGTAGACTAT	GTCATCCAATCAGAGAAGCCG
Atp23	GACTGCTCCCTTGTGAACGA	CGCACGCAAGTCTGATGATG
Atp5a	AGGCCTATCCTGGTGATGTG	CTTCATGGTACCTGCCACCT
Cd36	TGCTGGAGCTGTTATTGGTG	TGGGTTTTGCACATCAAAGA
Chop (Ddit3)	CCACCACACCTGAAAGCAGAA	AGGTGAAAGGCAGGGACTCA
Clpp	GCCATTCACTGCCCAATTCC	TGCTGACTCGATCACCTGTAG
Cpt1a	TATAACAGGTGGTTTGAC	CAGAGGTGCCCAATGATG
Cpt1b	TCGCAGGAGAAAACACCATGT	AACAGTGCTTGGCGGATGTG
Crif1	GAACGCTGGGAGAAAATTCA	ATAGTTCCTGGAAGCGAGCA
Fasn	CCCTTGATGAAGAGGGATCA	ACTCCACAGGTGGGAACAAG
Fgf21	AGATCAGGGAGGATGGAACA	TCAAAGTGAGGCGATCCATA
Fgfr1c	CCGTATGTCCAGATCCTGAAGA	GATAGAGTTACCCGCCAAGCA
G6pase	TCTGTCCCGGATCTACCTTG	GTAGAATCCAAGCGCGAAAC
Gck	CCCAGAAGGCTCAGAAGTTG	GCATCACCCTGAAGTTGGTT
Gdf15	GAGCTACGGGGTCGCTTC	GGGACCCCAATCTCACCT
Glut1	GCCCCCAGAAGGTTATTGA	CGTGGTGAGTGTGGTGGATG
Glut2	GGCTAATTTCAGGACTGGTT	TTTCTTTGCCCTGACTTCCT
Glut4	TGATTCTGCTGCCCTTCTGT	GGACATTGGACGCTCTCTCT
Grp78	GTGTGTGAGACCAGAACCGT	AGTCAGGCAGGAGTCTTAGG
Hspd1	GAGCTGGGTCCCTCACTCG	AGTCGAAGCATTTCTGCGGG

Htra2	TCCCCGGAGCCAGTACAAT	GAAAGGGTGCCGGTCTAGG
Immp1l	ATGACCCATGCACGCTTTGA	TCTGCTACCACCAGCCATAA
Immp2l	ACATGTGGGTTGAAGGCGAT	CCCAGAGAAACCGGTCCAAA
Klb (βKlotho)	GGAGGAGACCGTAAACTCGGGCTTA	ACGACCCGACGAGGGCTGTT
Lonp1	AGCCCTATGTTGGCGTCTTC	CCGGCTGATGTGAATCCTTCT
Pepck	GCATAACTAACCCCGAAGGCAAG	CATCCAGGCAATGTCATCGC
Pgc1α	TCACACCAAACCCACAGAAA	CTTGGGGTCATTTGGTGACT
Pparα	AGAAGTTGCAGGAGGGGATT	TTGAAGGAGCTTTGGGAAGA
Pparγ	TGCAGCTCAAGCTGAATCAC	ACGTGCTCTGTGACGATCTG
SpXbp1	CTGAGTCCGAATCAGGTGCAG	GTCCATGGGAAGATGTTCTGG
Srebp1	AAACTCAAGCAGGAGAACCTAAGTCT	GTCAGTGTGTCCTCCACCTCAGT
Srebp1a	GCGCCATGGACGAGCTG	TTGGCACCTGGGCTGCT
Srebp1c	ATCGCAAACAAGCTGACCTG	AGATCCAGGTTTGAGGTGGG
Srebp2	CAAGCTTCTAAAGGGCATCG	CACAAAGACGCTCAGGACAA
Tfam	TAGGCACCGTATTGCGTGAG	CAGACAAGACTGATAGACGAGGG
Tid1	GGAAGCAAGGATAGGCGAGA	GTTGACCGCTTTCCTCAGCAG
Ucp1	CACCTTCCCGCTGGACACT	CCCTAGGACACCTTTATACCTAATGG
Usp30	ACAAGCCCTTTTCTCCGCTT	GAACTGGGGAGTGATCGGTG

Table S4. Antibodies used for the Western blotting assays

Name	Source	Identifier
anti-CRIF1	Santa Cruz	Cat# sc-374122; RRID:AB_10917749
anti-NDUFA9	Abcam	Cat# ab14713; RRID:AB_301431
anti-SDHA	Cell Signaling	Cat# 5839S; RRID:AB_10707493
anti-UQCRC2	Abcam	Cat# ab14745; RRID:AB_2213640
anti-COX4	Santa Cruz	Cat# sc-58348; RRID:AB_2229944
anti-ATP5A	Invitrogen	Cat# 459240; RRID:AB_2532234
anti-TOM20 (FL-145)	Santa Cruz	Cat# sc-11415; RRID:AB_2207533
anti-β-actin	Sigma Aldrich	Cat# A2066; RRID:AB_476693
anti-OxPhos BN WB Antibody Cocktail	Invitrogen	Cat# 45-7999; RRID:AB_2533834
anti-COX I (1D6)	Santa Cruz	Cat# sc-58347; RRID:AB_2229888
anti-HSP60	Abcam	Cat# ab46798; RRID:AB_881444
anti-mtHSP70 (JG1)	Thermo Fisher Scientific	Cat# MA3-028; RRID:AB_325474
anti-TID1 (EPR12414)	Abcam	Cat# ab181024
anti-LONP1	Abcam	Cat# ab103809; RRID:AB_10858161
anti-CLPP (EPR12414)	Abcam	Cat# ab124822; RRID:AB_10975619
anti-CHOP (L63F7)	Cell Signaling	Cat# 2895; RRID:AB_2089254
anti-GRP78 (C50B12)	Cell Signaling	Cat# 3177; RRID:AB_2119845
anti-XBP1	Santa Cruz	Cat# sc-8015; RRID:AB_628449
anti-α-tubulin	Sigma Aldrich	Cat# T5168; RRID:AB_477579
anti-phospho-AKT-Ser473	Cell Signaling	Cat# 9271; RRID:AB_329825
anti-phospho-AKT-Thr308 (244F9)	Cell Signaling	Cat# 4056; RRID:AB_331163
anti-AKT	Cell Signaling	Cat# 9272; RRID:AB_329827
anti-phospho-GSK3α/β-Ser21/9	Cell Signaling	Cat# 9331; RRID:AB_329830
anti-GSK3β	Cell Signaling	Cat #9315S; RRID:AB_490890
anti-Caspase3	Cell Signaling	Cat# 9662; RRID:AB_331439
anti-PARP1 (F-2)	Santa Cruz	Cat# sc-8007; RRID:AB_628105
anti-PGC1α	Abcam	Cat# ab54481; RRID:AB_881987
anti-PGC1β	Abcam	Cat# ab61249; RRID:AB_946298

anti-mtTFA (E-16)	Santa Cruz	Cat# sc-30963; RRID:AB_2255881
anti-NRF2	Cell Signaling	Cat# 4399; RRID:AB_1950359
anti-UCP1	Abcam	Cat# ab10983; RRID:AB_2241462
anti-phospho-CREB-Ser133	Cell Signaling	Cat# 9191; RRID:AB_331606
anti-CREB (48H2)	Cell Signaling	Cat# 9197; RRID:AB_331277
anti-phospho-PKA-Thr197	Cell Signaling	Cat# 5661; RRID:AB_10707163
anti-PKA	Cell Signaling	Cat# 4782; RRID:AB_2170170
anti-ATGL	Cell Signaling	Cat# 2138s; RRID:AB_2167955
anti-GAPDH (14C10)	Cell Signaling	Cat# 2118; RRID:AB_561053

Reference

Caraux, G., and Pinloche, S. (2005). PermutMatrix: a graphical environment to arrange gene expression profiles in optimal linear order. Bioinformatics *21*, 1280-1281.

Huang da, W., Sherman, B.T., and Lempicki, R.A. (2009). Systematic and integrative analysis of large gene lists using DAVID bioinformatics resources. Nature protocols *4*, 44-57.

Kang, S.G., Yi, H.S., Choi, M.J., Ryu, M.J., Jung, S., Chung, H.K., Chang, J.Y., Kim, Y.K., Lee, S.E., Kim, H.W., et al. (2017). ANGPTL6 expression is coupled with mitochondrial OXPHOS function to regulate adipose FGF21. The Journal of endocrinology *233*, 105-118.

Kim, D., Langmead, B., and Salzberg, S.L. (2015). HISAT: a fast spliced aligner with low memory requirements. Nature methods *12*, 357-360.

Kwon, M.C., Koo, B.K., Moon, J.S., Kim, Y.Y., Park, K.C., Kim, N.S., Kwon, M.Y., Kong, M.P., Yoon, K.J., Im, S.K., et al. (2008). Crif1 is a novel transcriptional coactivator of STAT3. The EMBO journal *27*, 642-653.

Mi, H., Muruganujan, A., Casagrande, J.T., and Thomas, P.D. (2013). Large-scale gene function analysis with the PANTHER classification system. Nature protocols *8*, 1551-1566.

Mootha, V.K., Lindgren, C.M., Eriksson, K.F., Subramanian, A., Sihag, S., Lehar, J., Puigserver, P., Carlsson, E., Ridderstrale, M., Laurila, E., et al. (2003). PGC-1alpha-responsive genes involved in oxidative phosphorylation are coordinately downregulated in human diabetes. Nature genetics *34*, 267-273.

Subramanian, A., Tamayo, P., Mootha, V.K., Mukherjee, S., Ebert, B.L., Gillette, M.A., Paulovich, A., Pomeroy, S.L., Golub, T.R., Lander, E.S., et al. (2005). Gene set enrichment analysis: a knowledge-based approach for interpreting genome-wide expression profiles. Proceedings of the National Academy of Sciences of the United States of America *102*, 15545-15550.

Tan, C.M., Chen, E.Y., Dannenfelser, R., Clark, N.R., and Ma'ayan, A. (2013). Network2Canvas: network visualization on a canvas with enrichment analysis. Bioinformatics *29*, 1872-1878.

Yi, H.S., Lee, Y.S., Byun, J.S., Seo, W., Jeong, J.M., Park, O., Duester, G., Haseba, T., Kim, S.C., Park, K.G., et al. (2014). Alcohol dehydrogenase III exacerbates liver fibrosis by enhancing stellate cell activation and suppressing natural killer cells in mice. Hepatology *60*, 1044-1053.
